# Development of lysine-branched dendrimeric antimicrobial peptides targeting ESKAPE pathogens: broad-spectrum activity, biofilm eradication, and endotoxin neutralization

**DOI:** 10.3389/fmicb.2025.1702629

**Published:** 2026-02-23

**Authors:** S. Dinesh Kumar, Eun Young Kim, Naveen Kumar Radhakrishnan, Byambasuren Ganbaatar, Chul Won Lee, Sungtae Yang, Song Yub Shin

**Affiliations:** 1 Department of Cellular & Molecular Medicine, School of Medicine, Chosun University, Gwangju, Republic of Korea; 2 Department of Biomedical Sciences, School of Medicine, Chosun University, Gwangju, Republic of Korea; 3 Department of Chemistry, Chonnam National University, Gwangju, Republic of Korea; 4 Department of Microbiology, School of Medicine and Institute of Well-Aging Medicare & CSU GLAMP Project Group, Chosun University, Gwangju, Republic of Korea

**Keywords:** anti-inflammatory activity, antimicrobial peptides, cell selectivity, dendrimer, ESKAPE pathogens, LPS neutralization, proteolytic stability

## Abstract

Antimicrobial resistance (AMR) represents a pressing global health challenge, driving the urgent need for novel therapeutic agents with improved stability and selectivity. In this study, we present the rational design and synthesis of lysine-branched dendrimeric antimicrobial peptides (AMPs) based on short arginine/tryptophan-rich motifs (Du-6 and Lf-6), yielding dimeric and tetrameric architectures. Physicochemical analyses revealed a systematic increase in net charge and hydrophobicity with higher degrees of branching. Comparative biological evaluations demonstrated that dimeric peptides (di-Du-6 and di-Lf-6) achieved optimal broad-spectrum antibacterial activity against both Gram-positive and Gram-negative bacteria, including multidrug-resistant ESKAPE pathogens. These dimers maintained low hemolytic activity and exhibited therapeutic indices of up to 40. In contrast, despite their elevated charge density and tryptophan content, tetrameric peptides showed increased cytotoxicity, likely due to deeper membrane penetration into eukaryotic cells, thereby compromising selectivity. To overcome proteolytic degradation, D-enantiomeric dimers [(di-Du-6)*_D_* and (di-Lf-6)*_D_*] were synthesized. These retained potent antimicrobial efficacy, demonstrated complete resistance to trypsin digestion, and remained active under physiologically relevant conditions, including the presence of salts and serum. Beyond their antibacterial effects, the dimeric peptides effectively inhibited and eradicated biofilms formed by multidrug-resistant *Pseudomonas aeruginosa*, exhibited synergistic interactions with conventional antibiotics, and attenuated inflammatory responses by suppressing the production and expression of pro-inflammatory cytokines in LPS-stimulated macrophages. Furthermore, they neutralized endotoxins through direct binding and disaggregation of LPS aggregates. Collectively, these results establish dimeric peptides as multifunctional anti-infective agents, combining broad-spectrum antibacterial, antibiofilm, and anti-inflammatory activities. The enhanced proteolytic stability and selectivity of D-form dimers underscore their promise as next-generation therapeutics for combating multidrug-resistant infections and sepsis-associated inflammation.

## 1 Introduction

The escalating crisis of antimicrobial resistance (AMR) stands as one of the most urgent global health threats, with bacterial pathogens increasingly evading conventional antibiotics through mechanisms such as efflux pumps, enzymatic degradation, and biofilm formation (Brüssow, 2024). Recent estimates indicate that AMR is directly responsible for over 1.27 million deaths annually worldwide, and projections suggest that drug-resistant infections could lead to as many as 39 million fatalities by 2050 if left unaddressed (Ajulo and Awosile, 2024). In the United States alone, more than 2.8 million AMR-related infections occur each year, highlighting the critical need for innovative therapeutic approaches (Centers for Disease Control and Prevention [CDC], 2019). Of particular concern are the ESKAPE pathogens (*Enterococcus faecium, Staphylococcus aureus, Klebsiella pneumoniae, Acinetobacter baumannii, Pseudomonas aeruginosa,* and *Enterobacter* species), which are responsible for a substantial share of hospital-acquired infections and exhibit multidrug resistance (MDR), complicating treatment and contributing to elevated mortality rates (Roque-Borda et al., 2024; Lopes et al., 2022).

Antimicrobial peptides (AMPs) are naturally occurring cationic molecules produced by a wide range of organisms as part of their innate immune defense. They have emerged as promising alternatives to conventional antibiotics due to their broad-spectrum activity against bacteria, fungi, and viruses, rapid bactericidal action, and low tendency to induce resistance (Moretta et al., 2021). Typically, natural AMPs are short, cationic, and amphipathic, allowing them to interact with negatively charged microbial membranes and cause disruption or permeabilization, ultimately leading to cell lysis. In addition to membrane targeting, some AMPs can interfere with intracellular processes such as DNA replication and protein synthesis (Zhang et al., 2021). Their immunomodulatory functions, including cytokine regulation and endotoxin neutralization, further enhance their therapeutic potential in combating infections and associated inflammation (Roque-Borda et al., 2025). However, despite these advantages, the clinical application of natural AMPs faces several challenges, including rapid proteolytic degradation, poor serum stability, potential cytotoxicity to mammalian cells, and high production costs (Gonçalves et al., 2025; Mazumdar and Adam, 2021).

To overcome these limitations, a range of structural optimization strategies has been investigated, including sequence truncation, incorporation of non-natural amino acids, cyclization, substitution with D-amino acids, and multimerization through dendritic or branched architectures (Büyükkiraz and Kesmen, 2021; Little et al., 2025). Among these, lysine-based dendrimeric scaffolds offer precise control over valency, cationic charge density, and amphipathicity, thereby enhancing the local concentration of active motifs and improving resistance to proteolytic degradation compared to linear analogs (Tam et al., 2002; Paul et al., 2024; Liu et al., 2010). These dendrimeric designs also improve selectivity, minimizing hemolytic activity while retaining potent efficacy against biofilms and multidrug-resistant (MDR) strains (Frecer et al., 2004). Additionally, the incorporation of D-amino acid enantiomers provides exceptional resistance to proteases without compromising membrane-disruptive mechanisms, as their mirrored chirality avoids enzymatic recognition while maintaining essential physicochemical properties (Lu et al., 2020; Zhao et al., 2021). Collectively, these modifications have demonstrated promising results in enhancing serum stability and *in vivo* efficacy against ESKAPE pathogens (Jariyarattanarach et al., 2022; Boidin-Wichlacz et al., 2025).

In this study, we present a systematic approach to designing branched cationic AMPs based on two hexapeptide motifs: Du-6 (RKWKRW) and Lf-6 (RRWQWR), both enriched in arginine and tryptophan to facilitate strong electrostatic and hydrophobic interactions with bacterial membranes. Utilizing lysine-based branching, we constructed dimeric (di-Du-6 and di-Lf-6) and tetrameric (tet-Du-6 and tet-Lf-6) variants, enabling precise modulation of net charge (ranging from +8 to +20). This strategy was intended to harness multivalency for enhanced antimicrobial potency and selectivity. Among the synthesized peptides, the dimeric forms (di-Du-6 and di-Lf-6) demonstrated optimal broad-spectrum activity against both Gram-positive and Gram-negative bacteria, including multidrug-resistant ESKAPE pathogens (MICs: 15–18 μg/mL), while exhibiting significantly lower hemolytic and cytotoxic effects compared to their tetrameric counterparts. Owing to their superior therapeutic indices, we further developed D-enantiomeric analogs of the dimers, which preserved antimicrobial efficacy and showed complete resistance to tryptic degradation, along with improved tolerance to salt and serum conditions. Mechanistic investigations revealed that these branched dimers disrupt bacterial membranes via permeabilization and depolarization, and effectively neutralize lipopolysaccharide (LPS) through direct binding. Additionally, they strongly inhibit biofilm formation and exhibit synergistic effects when combined with conventional antibiotics, underscoring their multifunctional potential. Taken together, this work advances the rational design of next-generation AMPs and provides promising candidates for anti-infective therapeutics with a favorable balance of efficacy and safety.

## 2 Materials and methods

### 2.1 Materials

Details of the bacterial strains, mammalian cells, growth media, and other chemicals used in this study are provided in the Supplementary material.

### 2.2 Peptide synthesis

All peptides were synthesized via the 9-fluorenylmethoxycarbonyl (Fmoc) solid-phase method using Rink amide 4-methylbenzhydrylamine (MBHA) resin (0.56 mmol/g; Novabiochem, San Diego, CA, United States). Peptide purity was assessed by reversed-phase high-performance liquid chromatography (RP-HPLC) on an analytical Vydac C_18_ column (250 × 20 mm, 15 μm, 300 Å). The molecular masses of the purified peptides were confirmed using a triple-quadrupole mass spectrometer equipped with electrospray ionization liquid chromatography-mass spectrometry (ESI-LC–MS) (API2000, AB SCIEX).

### 2.3 Antimicrobial activity assay

The antimicrobial activity of the peptides against both standard and drug-resistant bacterial strains was assessed following the guidelines of the Clinical and Laboratory Standards Institute (CLSI, 2012 edition). A detailed description of the antimicrobial assay procedures is provided in the Supplementary material.

### 2.4 Hemolytic activity assay

The hemolytic activity of the peptides was assessed by quantifying hemoglobin release following the lysis of sheep red blood cells (sRBCs), as previously described (Kumar and Shin, 2020). A detailed protocol for the hemolytic assay is provided in the Supplementary material.

### 2.5 Toxicity against RAW 264.7 cells

The cytotoxicity of the peptides against RAW 264.7 cells was evaluated using the MTT dye reduction assay, as previously described (Kumar and Shin, 2020). A detailed protocol is provided in the Supplementary material.

### 2.6 Steady-state tryptophan fluorescence and acrylamide quenching assay

Tryptophan fluorescence spectra of dimeric and tetrameric peptides were recorded in 10 mM Tris–HCl buffer (pH 7.4), both in the absence and presence of small unilamellar vesicles (SUVs) composed of PC/Chol (10:1, w/w) or PE/PG (7:3, w/w). Excitation was set at 295 nm, and emission spectra were collected from 300 to 400 nm, as previously described (Azmi, 2013). The wavelength corresponding to the maximum fluorescence intensity (*λ*_max_) was determined under each condition. All experiments were performed in triplicate, and the data were averaged. Acrylamide quenching of tryptophan fluorescence was conducted according to established protocols. The resulting data were analyzed using the Stern–Volmer equation, and the Stern–Volmer quenching constant (*K*_SV_) was calculated as previously reported (Zhu et al., 2006).

### 2.7 Trypsin digestion assay

Peptide stability against proteolytic degradation was evaluated using a trypsin digestion assay, following the previously described protocol (Kim et al., 2023). Detailed experimental procedures are provided in the Supplementary material.

### 2.8 Cytoplasmic membrane depolarization assay

Cytoplasmic membrane depolarization in bacterial cells was assessed using the potential-sensitive fluorescent dye 3,3-dipropylthiadicarbocyanine iodide (diSC_3_-5), following previously established methods (Kim et al., 2023). A detailed protocol is provided in the Supplementary material.

### 2.9 Flow cytometry analysis

Peptide-induced membrane damage was quantified by flow cytometry, following the previously described method (Kumar et al., 2024a). Detailed experimental procedures are provided in the Supplementary material.

### 2.10 Outer membrane permeabilization assay

Outer membrane permeabilization in Gram-negative *E. coli* (KCTC 1682) was assessed by measuring the uptake of the fluorescent dye *N*-phenyl-1-naphthylamine (NPN), as previously described (Kumar and Shin, 2020). Detailed procedures are provided in the Supplementary material.

### 2.11 Antibiofilm activity assay

Biofilm inhibition and eradication were evaluated by determining the minimum biofilm inhibitory concentration (MBIC) and minimum biofilm eradication concentration (MBEC), as previously reported (Harrison et al., 2010). A complete assay description is available in the Supplementary material.

### 2.12 Confocal laser-scanning microscopy (CLSM)

Biofilm eradication by peptides against preformed multidrug-resistant *Acinetobacter baumannii* (MDRAB 329–53) biofilms was visualized using CLSM with LIVE/DEAD (SYTO9/PI) staining, as described earlier (Kim et al., 2023). Experimental details are provided in the Supplementary material.

### 2.13 Checkerboard assay

Synergistic interactions between peptides and conventional antibiotics were analyzed using a checkerboard assay, following the method described earlier (Qu et al., 2017). A detailed protocol is included in the Supplementary material.

### 2.14 Quantification of inflammatory cytokine release

Inhibition of tumor necrosis factor-*α* (TNF-α), interleukin-6 (IL-6), and monocyte chemoattractant protein-1 (MCP-1) production in LPS-stimulated RAW 264.7 cells was quantified using ELISA, as previously described (Kumar et al., 2024b). A detailed description is provided in the Supplementary material.

### 2.15 Cytokine mRNA expression analysis (RT-PCR)

The effects of the peptides on the mRNA expression levels of TNF-α, IL-6, and MCP-1 in LPS-stimulated RAW 264.7 cells were determined using Reverse Transcription-Polymerase Chain Reaction (RT-PCR). GAPDH was used as a housekeeping gene control. A detailed description is provided in the Supplementary material.

### 2.16 LPS neutralization assay

The LPS-neutralizing activity of peptides was assessed using the BODIPY-TR-cadaverine (BC) displacement assay, following the method described (Wood et al., 2004). Detailed procedures are provided in the Supplementary material.

### 2.17 Dissociation of LPS-FITC aggregates

Disaggregation of fluorescein isothiocyanate (FITC)-labeled LPS oligomers was evaluated according to previously established protocols (Rosenfeld et al., 2006; Haas et al., 2000). Full experimental details are available in the Supplementary material.

### 2.18 Effect of peptides on LPS-binding to macrophages and receptor-bound LPS

The ability of peptides to inhibit FITC–LPS binding to RAW 264.7 macrophages or to neutralize receptor-bound LPS was examined as previously reported (Kim et al., 2023; Rosenfeld et al., 2006). A detailed description of the methodology is provided in the Supplementary material.

### 2.19 Statistical analysis

Statistical analyses were performed using the Student’s *t*-test for comparisons between two groups and one-way analysis of variance (ANOVA) followed by Tukey’s multiple comparison test for comparisons among more than two groups. Calculations were conducted using SPSS version 22 (SPSS Inc., IL, United States), GraphPad Prism 8, SigmaPlot V12, and Origin 2018. Data are presented as mean ± standard deviation (SD), and statistical significance was defined as *p* < 0.05. Group comparisons were made using unpaired, two-tailed *t*-tests. Significance levels are indicated as *(*p* < 0.05), **(*p* < 0.01), and ***(*p* < 0.001).

## 3 Results

### 3.1 Peptide design

To systematically assess the impact of dendrimerization on antimicrobial activity and selectivity, we designed a series of cationic peptides based on two amphipathic hexapeptide motifs: Du-6 (RKWKRW). To generate multimeric branched peptides, we employed a lysine-based scaffold, producing dimeric (di-Du-6 and di-Lf-6) and tetrameric (tet-Du-6 and tet-Lf-6) peptides (Table 1). Dimeric peptides were synthesized by conjugating two hexapeptide motifs to a single lysine (K) core, while tetrameric peptides utilized a di-lysine core (K₂K) to anchor four motifs.

**Table 1:** Amino acid sequences and physicochemical properties of the peptides.

Peptides	Amino acid sequence	Net charge	*R*_t_^a^	MS analysis^b^		
				z	m/z calculated	m/z found
Du-6	RKWKRW-βA-NH_2_	+5	13.82	[M + 2H]^2+^ [M + 3H]^3+^	515.6 344.8	516.1 344.7
Lf-6	RRWQWR-βA-NH_2_	+4	13.05	[M + 2H]^2+^ [M + 3H]^2+^	529.6 353.4	530.1 353.9
di-Du-6	(RKWKRW)_2_-K-βA-NH_2_	+10	15.54	[M + 3H]^3+^ [M + 4H]^4+^	700.5 525.6	700.8 526.1
di-Lf-6	(RRWQWR)_2_-K-βA-NH_2_	+8	13.55	[M + 3H]^3+^ [M + 4H]^4+^	719.18 539.63	719.5 540.0
tet-Du-6	(RKWKRW)_4_-K_2_K-βA-NH_2_	+20	18.48	[M + 3H]^3+^ [M + 4H]^4+^	1413.1 1060.1	1413.8 1060.8
tet-Lf-6	(RRWQWR)_4_-K_2_K-βA-NH_2_	+16	17.72	[M + 3H]^3+^ [M + 4H]^4+^	1450.4 1088.0	1450.9 1088.9

^a^Retention time (*R*_t_) was determined by analytical RP-HPLC on a C_18_ column (5 mm; 4.6 mm × 250 mm; Vydac) using a gradient of buffer B (0.05% TFA in CH_3_CN/H_2_O 90:10 v/v) in buffer A (0.05 %TFA in H_2_O) for 60 min with a flow rate of 1.0 mL/min.

^b^Molecular mass was determined using matrix-assisted laser desorption/ionization (MALDI)-time-of-flight (TOF)-mass spectrometry (MS).

Du-6 exhibited a net charge of +5, whereas Lf-6 carried a charge of +4. Dimerization increased the net charge to +10 for di-Du-6 and +8 for di-Lf-6, while tetramerization further elevated the charge to +20 and +16, respectively (Table 1). Notably, the lysine core amino groups were fully acylated during synthesis and therefore did not contribute to the overall charge. This systematic enhancement of valency and charge density allowed us to investigate how branching architecture influences antimicrobial potency, cytotoxicity, and selectivity.

### 3.2 Characterization of peptides

All peptides were successfully synthesized with high purity (>95%), as verified by reverse-phase high-performance liquid chromatography (RP-HPLC) (Supplementary Figure S1) and precise mass determination using electrospray ionization mass spectrometry (ESI-MS; Supplementary Figure S2). The experimentally measured molecular weights closely matched the theoretical values, confirming both the successful synthesis and accurate assembly of the branched peptide architectures (Table 1).

As designed, all synthetic peptides exhibited a cationic nature, with tetrameric constructs showing higher charge states due to their increased basicity. Retention times increased progressively from 13.82 min for Du-6 to 17.72 min for tet-Du-6, indicating enhanced hydrophobicity associated with greater branching and molecular size (Table 1).

### 3.3 Antimicrobial activity and cell cytotoxicity

**Figure 1:**
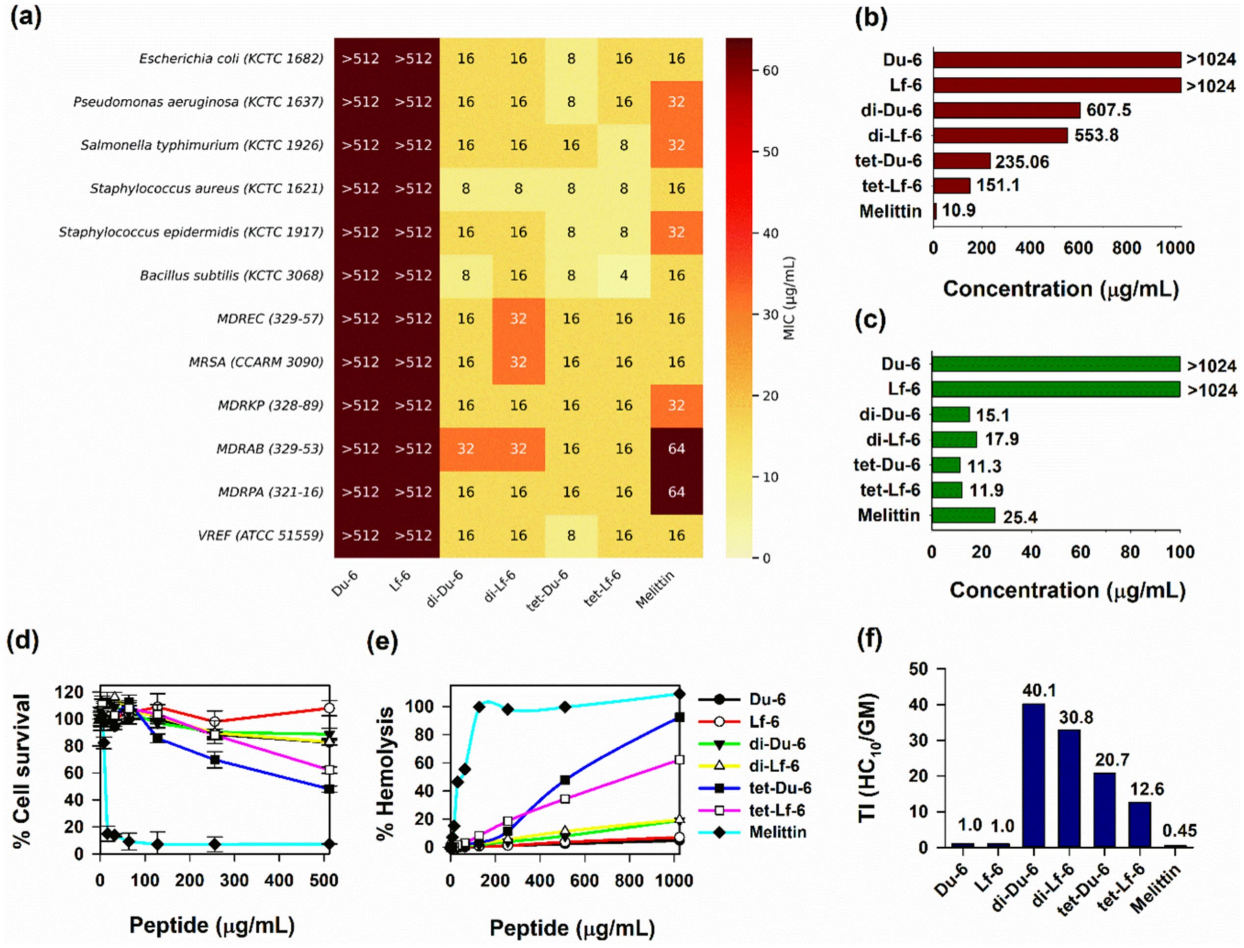
**(a)** Heatmap of minimum inhibitory concentrations (MICs) of the Du-6, Lf-6, di-Du-6, di-Lf-6, tet-Du-6, and tet-Lf-6 against different bacterial strains. MICs were determined as the lowest concentration of the peptides that inhibited bacterial growth. **(b)** HC_10_ of the peptide concentration that caused 10% hemolysis of sheep red blood cells (sRBCs). **(c)** Geometric mean (GM) of MIC values against bacterial strains. **(d)** Cytotoxicity of peptides against murine macrophage RAW 264.7 cells. **(e)** Hemolytic activity against sRBCs. **(f)** The therapeutic index (TI) of the peptides is the ratio of the HC_10_ value (μg/mL) over GM (μg/mL).

The antimicrobial efficacy of the synthesized peptides was assessed against a panel of Gram-positive and Gram-negative bacteria, including multidrug-resistant (MDR) ESKAPE pathogens. As shown in the heatmap (Figure 1a), both monomeric peptides (Du-6 and Lf-6) exhibited minimal activity, with minimum inhibitory concentration (MIC) values exceeding 512 μg/mL across all tested strains. In contrast, branched analogs demonstrated potent and broad-spectrum antimicrobial activity. Among them, the dimeric peptides di-Du-6 and di-Lf-6 showed the most pronounced activity, with geometric mean (GM) MIC values of 15.1 and 18.0 μg/mL, respectively (Figure 1c). Despite their higher charge density, the antimicrobial potency of tetrameric peptides (GM MIC: 11.31–11.99 μg/mL) was not significantly greater than that of the dimers.

Peptide cytotoxicity toward mammalian cells was evaluated using murine RAW 264.7 macrophage viability assays (Figure 1d) and hemolytic activity against sheep red blood cells (sRBCs) (Figure 1e). Monomeric peptides (Du-6 and Lf-6) showed no detectable hemolysis at concentrations up to 1,024 μg/mL. Among the branched peptides, dimers exhibited low hemolytic activity, with HC_10_ values (Figure 1b) of 607.59 μg/mL (di-Du-6) and 553.83 μg/mL (di-Lf-6). In contrast, tetrameric peptides tet-Du-6 and tet-Lf-6 displayed increased hemolysis, with HC_10_ values of 235.06 μg/mL and 151.09 μg/mL, respectively. Cytotoxicity toward RAW 264.7 cells followed a similar pattern: all peptides maintained >80% cell viability at concentrations up to 128 μg/mL, while tetramers showed moderate toxicity at higher doses (Figure 1d). Melittin, used as a positive control, induced pronounced hemolysis (HC_10_: ~11 μg/mL) and significant macrophage toxicity even at low concentrations. Collectively, these findings indicate that while both dimeric and tetrameric peptides possess antimicrobial activity, dimers offer superior cell selectivity due to their markedly reduced cytotoxicity toward mammalian cells.

### 3.4 Therapeutic index of branched peptides

To assess the balance between antimicrobial potency and mammalian cell selectivity, the therapeutic index (TI) was calculated as the ratio of HC_10_ to the geometric mean MIC (Figure 1f). Monomeric peptides exhibited negligible TI values (<1.0), reflecting their lack of antimicrobial activity. In contrast, dimeric peptides demonstrated markedly improved therapeutic profiles, with TI values of 40.1 for di-Du-6 and 30.8 for di-Lf-6, indicating strong antimicrobial efficacy coupled with minimal hemolytic toxicity. Although tetrameric peptides showed slightly enhanced antimicrobial potency based on MIC values, their increased cytotoxicity significantly diminished their therapeutic indices, yielding TI values of 20.8 for tet-Du-6 and 12.6 for tet-Lf-6. As expected, melittin, used as a positive control exhibited a poor TI of 0.45, consistent with its pronounced hemolytic activity. These findings underscore the favorable therapeutic window of dimeric constructs, which achieve potent antimicrobial effects while maintaining low cytotoxicity toward mammalian cells.

### 3.5 Intrinsic tryptophan fluorescence in membrane-mimicking environments

**Figure 2:**
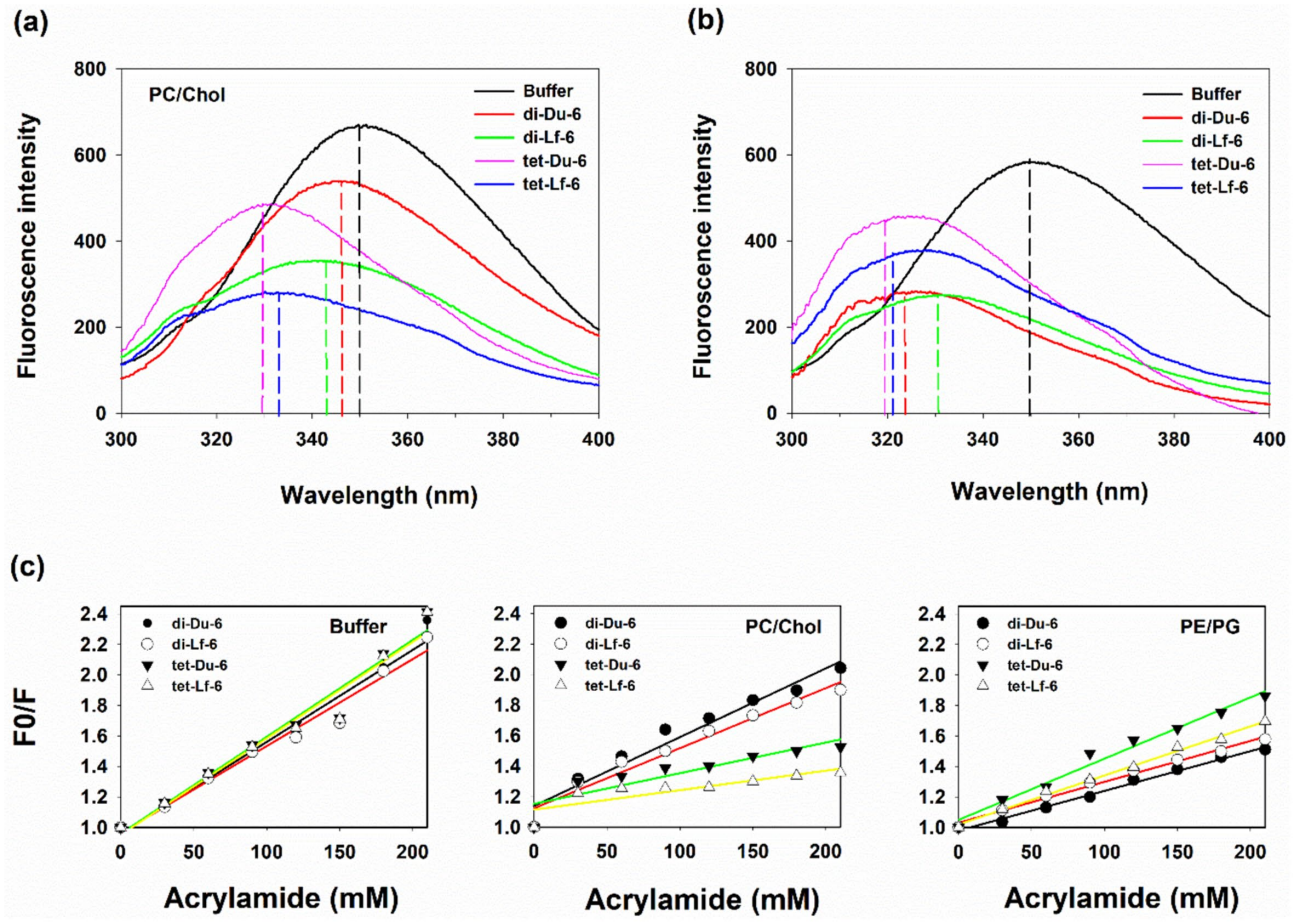
Intrinsic tryptophan fluorescence studies of dimer and tetramer analogs. **(a)** Fluorescence emission spectra of di-Du-6, di-Lf-6, tet-Du-6, and tet-Lf-6 in the presence of PC/Chol small unilamellar vesicles (SUVs), mimicking eukaryotic cell membranes. **(b)** Fluorescence emission spectra in the presence of PE/PG SUVs, mimicking bacterial cell membranes. **(c)** Stern–Volmer plots of tryptophan quenching by acrylamide for the peptides in aqueous buffer, PC/Chol SUVs, and PE/PG SUVs. Excitation wavelength was 295 nm, and all measurements were performed at 25 °C in phosphate-buffered saline (pH 7.4). Data represent the mean of three independent experiments.

Intrinsic tryptophan fluorescence was employed to assess the insertion depth of branched peptides within bilayer environments. Fluorescence emission spectra were recorded for both dimeric (di-Du-6 and di-Lf-6) and tetrameric (tet-Du-6 and tet-Lf-6) peptides in aqueous buffer (pH 7.4) and in the presence of small unilamellar vesicles (SUVs) that mimic either eukaryotic (PC/Chol, 10:1 w/w) or bacterial (PE/PG, 7:3 w/w) membranes (Figures 2a,b). In aqueous buffer, all peptides exhibited emission maxima (*λ*_max_) around 350 nm, indicating that the tryptophan residues were exposed to a polar environment. Upon addition of PC/Chol SUVs, tetrameric peptides showed a pronounced blue shift in *λ*_max_ to 325–330 nm, suggesting deeper insertion into the hydrophobic core of the eukaryotic membrane mimics. This deeper insertion is consistent with their elevated hemolytic activity. In contrast, dimeric peptides exhibited a more modest shift to approximately 345 nm, indicative of surface-level association rather than full membrane penetration, aligning with their lower cytotoxicity. When exposed to anionic PE/PG SUVs, both dimeric and tetrameric peptides demonstrated intermediate blue shifts (*λ*_max_ ≈ 320–330 nm). Notably, tetrameric peptides showed slightly greater shifts and enhanced fluorescence intensity compared to their dimeric counterparts, reflecting stronger interactions with bacterial membrane mimics.

### 3.6 Acrylamide quenching of tryptophan fluorescence

To further elucidate the membrane localization of peptides, acrylamide quenching of tryptophan fluorescence was performed in aqueous buffer and in the presence of PC/Chol or PE/PG SUVs (Figure 2c). Acrylamide, a neutral and water-soluble quencher, does not engage in electrostatic interactions with the negatively charged headgroups of anionic phospholipids (Sood et al., 2008). Quenching efficiency was quantified using Stern–Volmer analysis under three conditions: buffer alone, PC/Chol SUVs, and PE/PG SUVs. In buffer, the Stern–Volmer quenching constants (K*_SV_*) were relatively high (K*_SV_ =* 2.2–2.4 M^−1^), indicating that tryptophan residues were solvent-exposed. In the presence of PC/Chol SUVs, dimeric peptides remained efficiently quenched (K*_SV_ =* 1.8–2.0 M^−1^), suggesting that their tryptophan residues were still accessible to the aqueous phase and likely positioned near the membrane surface. In contrast, tetrameric peptides exhibited significantly reduced quenching (K*_SV_ =* 1.2–1.4 M^−1^), consistent with deeper insertion of tryptophan residues into the hydrophobic core of the bilayer. With PE/PG SUVs, both dimeric and tetrameric peptides showed similarly reduced quenching efficiencies (K*_SV_ =* 1.4–1.8 M^−1^ for tetramers and K*_SV_ =* 1.4–1.6 M^−1^ for dimers), indicating partial burial of tryptophan residues within the negatively charged phospholipid membranes. Collectively, these findings suggest that tetrameric peptides preferentially partition into zwitterionic eukaryotic membranes, while dimeric peptides associate more superficially. This behavior correlates with the lower hemolytic activity and higher cell selectivity observed for the dimeric peptides.

Given their potent antimicrobial activity, low cytotoxicity, and favorable therapeutic indices, the dimeric peptides di-Du-6 and di-Lf-6 were identified as the most promising candidates. To further improve their resistance to proteolytic degradation while preserving bioactivity, D-enantiomeric analogs [(di-Du-6)*_D_* and (di-Lf-6)*_D_*] were synthesized for subsequent investigations (Supplementary Table S1).

### 3.7 Effect of salts and serum on antimicrobial activity

The antimicrobial activities of di-Du-6, di-Lf-6, and their D-enantiomeric counterparts were assessed in the presence of physiologically relevant salts and 25% human serum against *Staphylococcus aureus* and *Escherichia coli* (Table 2). For the L-form dimers, the addition of monovalent (Na^+^, K^+^, NH_4_^+^), divalent (Mg^2+^, Ca^2+^), or trivalent (Fe^3+^) cations had minimal to no impact on activity against *E. coli*. In contrast, modest reductions in activity were observed against *S. aureus*, particularly with di-Lf-6. Human serum exerted a more pronounced effect, resulting in approximately 2–4-fold increases in MIC values, suggesting partial inactivation due to interactions with serum proteins. Conversely, the D-enantiomeric peptides (di-Du-6)*_D_* and (di-Lf-6)*_D_* maintained full antimicrobial activity under both salt-rich and serum-containing conditions. Their MIC values remained consistent across all ionic environments and in 25% human serum, demonstrating superior resilience compared to their native L-form counterparts. These results indicate that D-enantiomerization enhances antimicrobial efficacy under physiologically challenging conditions.

**Table 2:** The MIC in the presence of physiological salts and human serum against *Escherichia coli* (KCTC 1682) and *Staphylococcus aureus* (KCTC 1621).

Compounds	Control	NaCl 150 mM	KCl 4.5 mM	NH_4_Cl 4 microM	MgCl_2_ 1 mM	CaCl_2_ 2.5 mM	FeCl_3_ 6 microM	Human serum (25%)
*Staphylococcus aureus* (KCTC 1621)								
di-Du-6	8	8	8	8	8	8	16	16
di-Lf-6	8	16	16	16	16	16	32	16
(di-Du-6)*_D_*	8	8	8	8	8	8	8	8
(di-Lf-6)*_D_*	8	8	8	8	8	8	8	8
*Escherichia coli* (KCTC 1682)								
di-Du-6	16	16	16	16	16	16	16	16
di-Lf-6	16	16	16	16	16	16	16	32
(di-Du-6)*_D_*	8	8	8	16	8	8	16	8
(di-Lf-6)*_D_*	16	8	8	16	16	8	16	16

The control MICs were determined in the absence of salts or human serum.

### 3.8 Proteolytic stability against trypsin

**Figure 3:**
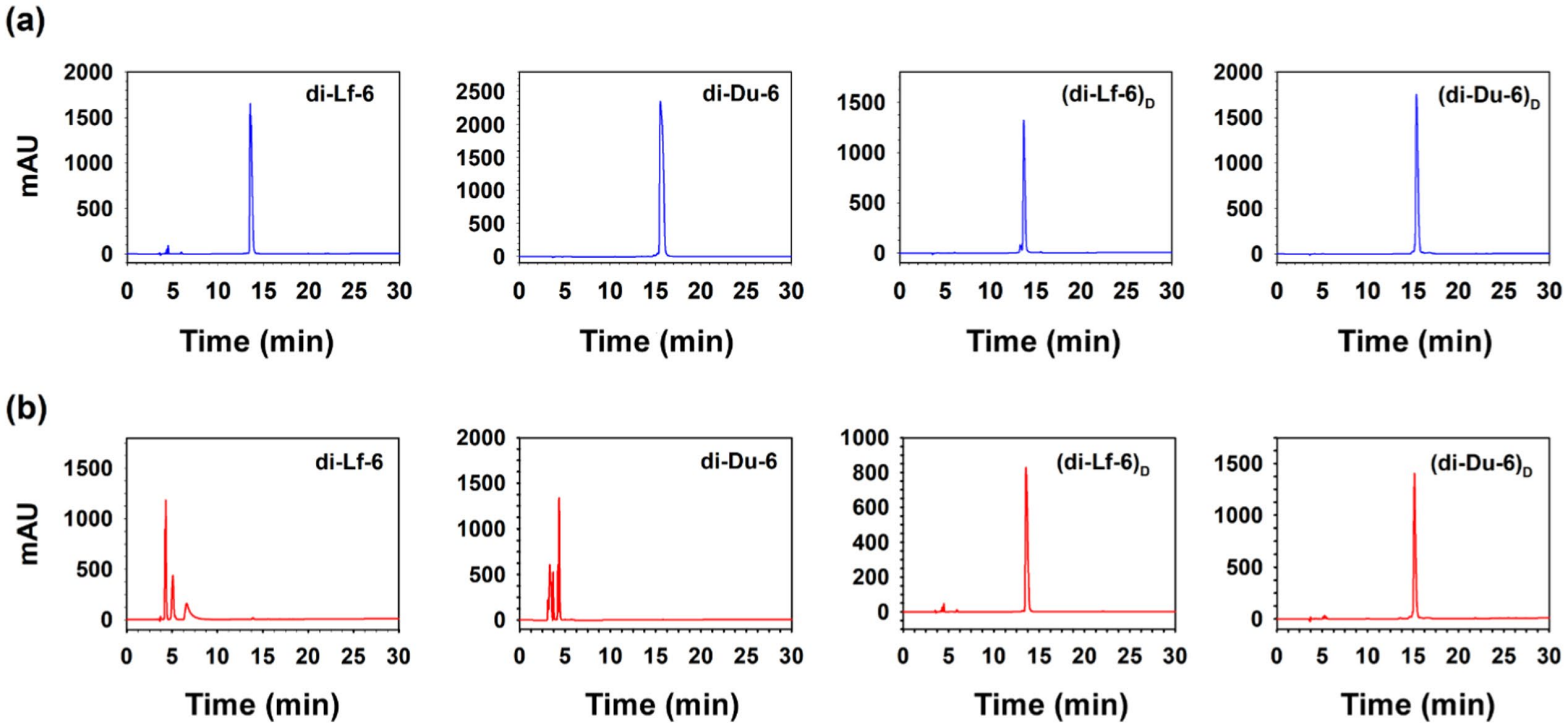
Reverse-phase high-performance liquid chromatography (RP-HPLC) profiles of tryptic digestion products of di-Du-6, di-Lf-6, (di-Du-6)*_D_*, and (di-Lf-6)*_D_*. **(a)** 0 h incubation with trypsin (control). **(b)** 24 h incubation with trypsin.

To evaluate the susceptibility of the peptides to enzymatic degradation, di-Du-6, di-Lf-6, and their D-enantiomeric analogs were incubated with trypsin for 24 h and subsequently analyzed using RP-HPLC (Figure 3). The L-form dimers were rapidly degraded, as evidenced by the complete disappearance of intact peptide peaks after 24 h, confirming their vulnerability to proteolytic cleavage. In contrast, the D-form peptides [(di-Du-6)*_D_* and (di-Lf-6)*_D_*] remained fully intact under the same conditions, with no detectable degradation products. These findings demonstrate that substitution with D-amino acids confers complete resistance to trypsin-mediated proteolysis, thereby markedly enhancing peptide stability under physiologically relevant conditions.

### 3.9 Mechanistic evaluation of branched peptides

Cationic AMPs typically exert bactericidal effects by disrupting bacterial membranes through electrostatic and hydrophobic interactions, ultimately leading to membrane destabilization and cell death (Luo and Song, 2021). To investigate the antimicrobial mechanism of the branched peptides, membrane depolarization, outer membrane permeability, and membrane integrity assays were conducted using representative Gram-positive (*S. aureus*) and Gram-negative (*E. coli*) strains.

### 3.10 Membrane depolarization and outer membrane permeability

**Figure 4:**
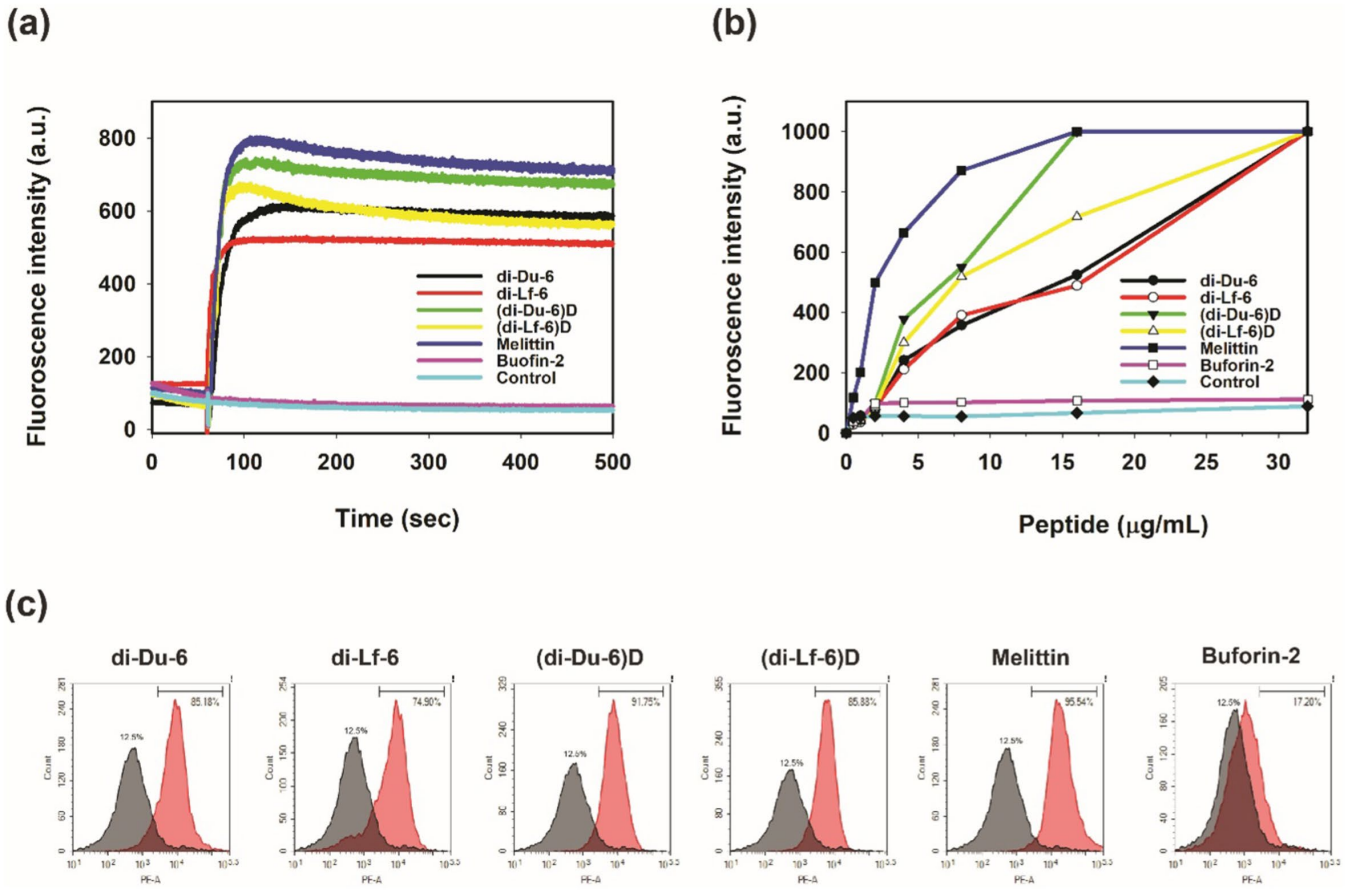
Bactericidal mechanism of di-Du-6, di-Lf-6, (di-Du-6)*_D_*, and (di-Lf-6)_*D*._ **(a)** Peptide-induced membrane depolarization measured with DiSC_3_(5) against intact *S. aureus* (KCTC 1621). **(b)** Outer membrane permeability. Membrane uptake of 1-N-phenylnaphthylamine (NPN) by *E. coli* KCTC 1682 in the presence of increasing concentrations of peptides. **(c)** Inner membrane integrity assessment by flow cytometric analysis of *E. coli* (KCTC 1682) cells treated with peptides at 1 × MIC concentrations. The gray shaded area in all histograms represents the fluorescence of the untreated control cells (12.5% PI-positive). The red shaded area represents the fluorescence of the peptide-treated cells.

Cytoplasmic membrane depolarization was evaluated using the potential-sensitive dye diSC_3_-5. At a concentration of 1 × MIC, di-Du-6, di-Lf-6, and their D-enantiomeric counterparts induced rapid depolarization in *S. aureus*, comparable to the effect of melittin, as indicated by increased fluorescence intensity (Figure 4a). In contrast, buforin-2, an intracellular-targeting control peptide, showed no measurable effect. Outer membrane permeabilization in *E. coli* was further assessed using NPN uptake. All four dimeric peptides triggered a dose-dependent increase in NPN fluorescence, with the D-enantiomers demonstrating greater permeabilizing activity at 1 × MIC compared to their L-form counterparts (Figure 4b). NPN is a fluorescent probe that penetrates the hydrophobic core of the bacterial outer membrane upon disruption by peptides, resulting in a marked increase in fluorescence intensity. As shown in Figure 4b, similar to melittin, all dimeric peptides caused a dose-dependent enhancement in outer membrane permeability in *E. coli*. Notably, (di-Du-6)*_D_* and (di-Lf-6)*_D_* exhibited significantly higher permeability at 1 × MIC than di-Du-6 and di-Lf-6, underscoring the enhanced membrane-disruptive potential of the D-enantiomeric forms.

### 3.11 Inner membrane integrity assessment

The integrity of the bacterial inner membrane was further evaluated using propidium iodide (PI) uptake combined with flow cytometry analysis (Kim et al., 2023). Untreated *E. coli* cells and those treated with buforin-2 exhibited minimal PI staining (12.5 and 17.2%, respectively), indicative of intact membranes (Figure 4c). In contrast, treatment with dimeric peptides at 1 × MIC resulted in substantial PI influx, with 75–92% of cells becoming PI-positive, comparable to the effect observed with melittin (95.5%). These findings confirm that the bactericidal activity of the dimeric branched peptides is primarily mediated by membrane disruption rather than intracellular targeting.

Taken together, the results from membrane depolarization, outer membrane permeability, and PI uptake assays demonstrate that both L- and D-form dimers exert their antimicrobial effects through direct disruption of bacterial membranes. Notably, the D-enantiomeric peptides exhibited slightly enhanced potency.

### 3.12 Biofilm inhibition and eradication by dimeric peptides

**Figure 5:**
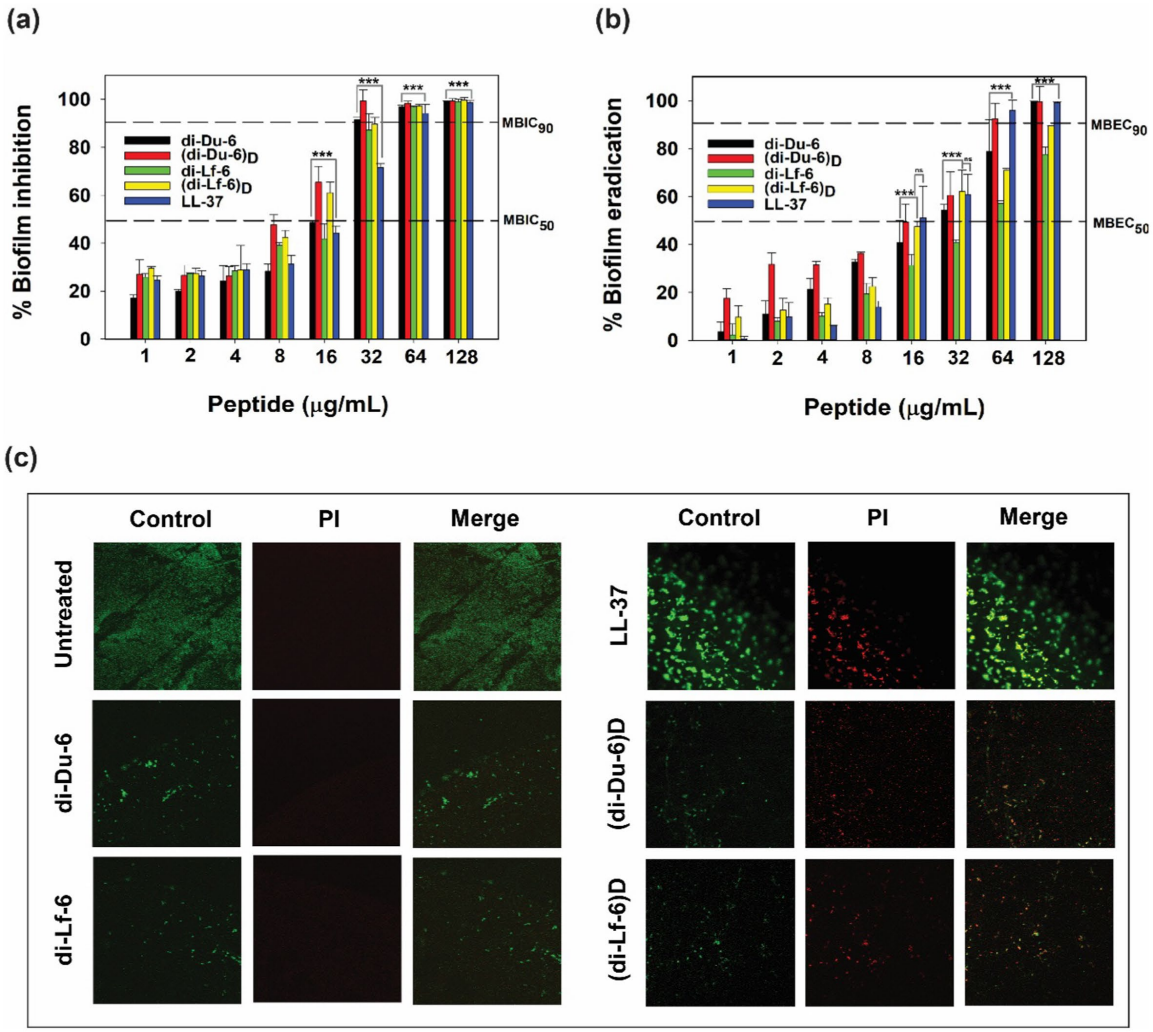
Antibiofilm activity of di-Du-6, di-Lf-6, (di-Du-6)*_D,_* and (di-Lf-6)*_D_* against MDRPA (321–16) biofilms. **(a)** Biofilm inhibition. **(b)** Biofilm eradication. **(c)** Confocal laser scanning microscopy (CLSM) images of peptide-treated mature MDRPA biofilms. Significance levels are indicated as *p < 0.05, **p < 0.01, ***p < 0.001, and ns for not significant.

The ability of dimeric peptides and their D-enantiomeric counterparts to inhibit and eradicate biofilms was assessed using multidrug-resistant *Pseudomonas aeruginosa* (MDRPA) (321–16). Minimum biofilm inhibitory concentrations (MBIC) and minimum biofilm eradication concentrations (MBEC) were determined using the Calgary Biofilm Device (CBD) (Ceri et al., 1999), while structural disruption of biofilms was further analyzed via confocal laser scanning microscopy (CLSM). Both di-Du-6 and di-Lf-6 exhibited concentration-dependent inhibition of biofilm formation, with MBIC_50_ values ranging from 16 to 32 μg/mL and MBIC_90_ values from 32 to 64 μg/mL (Figure 5a). Their D-enantiomeric analogs demonstrated equal or superior potency, with (di-Du-6)*_D_* showing the lowest MBIC_50_ at 8 μg/mL. Eradication of preformed biofilms required higher peptide concentrations, with MBEC values ranging from 64 to 128 μg/mL (Figure 5b). Notably, the D-form peptides were more effective, achieving biofilm eradication at concentrations 2–4 times lower than their L-form counterparts and comparable to the human cathelicidin LL-37. Confocal laser scanning microscopy (CLSM) of SYTO-9/PI-stained biofilms further confirmed the substantial reduction in biofilm mass and increased bacterial cell death following peptide treatment. Untreated MDRPA biofilms exhibited dense, intact microcolonies with strong green fluorescence, indicative of viable cells. In contrast, treatment with dimeric peptides resulted in pronounced biofilm disruption and decreased biomass (Figure 5c). Among the tested analogs, (di-Du-6)*_D_* induced the most significant disaggregation of mature biofilms, leaving only sparse residual structures at a concentration of 32 μg/mL. Collectively, these findings demonstrate that both L- and D-form dimers effectively inhibit biofilm formation and facilitate the disassembly of established MDRPA biofilms, with D-enantiomers showing superior efficacy in both prophylactic and therapeutic contexts.

### 3.13 Synergistic antimicrobial effect with conventional antibiotics

To determine whether branched dimeric peptides enhance the efficacy of conventional antibiotics, checkerboard broth microdilution assays were conducted against MDRPA (321–16). Fractional inhibitory concentration indices (FICI) were calculated to evaluate drug–peptide interactions, where FICI ≤ 0.5 indicates synergy, 0.5 < FICI ≤ 1.0 indicates additive effects, and FICI > 1.0 indicates indifference. Dimeric peptides exhibited synergistic interactions with chloramphenicol (CPN) across all tested combinations, with FICI values ranging from 0.25 to 0.38 (Table 3). Notably, both L- and D-forms of di-Du-6 and di-Lf-6 reduced the MIC of CPN by up to 8-fold, while concurrently lowering their own MICs by 2–4-fold, indicating mutual potentiation. In the case of ciprofloxacin (CIP), synergy was observed with di-Lf-6 and both D-form peptides (FICI: 0.37–0.38), whereas di-Du-6 showed an additive effect (FICI: 0.63). Conversely, combinations with oxacillin (OXA) resulted in additive interactions only (FICI: 0.75–1.0), with no evidence of synergism. These results suggest that the antimicrobial activity of dimeric peptides can be significantly enhanced when used in combination with specific antibiotics, particularly CPN and CIP. The pronounced synergy observed with D-form dimers underscores their potential as adjuvant agents to restore or augment the efficacy of conventional antibiotics against multidrug-resistant pathogens.

**Table 3:** Fractional inhibitory concentration index (FICI) of the peptides in combination with conventional antibiotics against MDRPA (321–16).

Combinations		MIC_A_ (μg/mL)	[A] (μg/mL)	FIC_A_	MIC_B_ (μg/mL)	[B] (μg/mL)	FIC_B_	FICI	Combination effect
Antibiotics	Peptides								
CPN	di-Du-6	4,096	512	0.125	16	4	0.25	0.375	Synergy
	di-Lf-6	4,096	512	0.125	32	8	0.25	0.375	Synergy
	(di-Du-6)*_D_*	4,096	512	0.125	32	4	0.125	0.25	Synergy
	(di-Lf-6)*_D_*	4,096	512	0.125	32	8	0.25	0.375	Synergy
CIP	di-Du-6	1,024	128	0.125	16	8	0.5	0.625	Additive
	di-Lf-6	1,024	256	0.25	32	4	0.125	0.375	Synergy
	(di-Du-6)*_D_*	1,024	128	0.125	32	8	0.25	0.375	Synergy
	(di-Lf-6)*_D_*	1,024	128	0.125	32	8	0.25	0.375	Synergy
OXA	di-Du-6	4,096	1,024	0.25	16	8	0.5	0.75	Additive
	di-Lf-6	4,096	2048	0.5	32	16	0.5	1	Additive
	(di-Du-6)*_D_*	4,096	1,024	0.25	32	16	0.5	0.75	Additive
	(di-Lf-6)*_D_*	4,096	2048	0.5	32	16	0.5	1	Additive

CPN, chloramphenicol; CIP, ciprofloxacin; OXA, oxacillin.

MIC_A_, MIC antibiotic A alone; [A], MIC of antibiotic A in combination. FIC_A_ = [A]/MIC_A._

MIC_B_, MIC of peptide B alone; [B], MIC of peptide B in combination. FIC_B_ = [B]/MIC_B._

FICI = FIC_A_ + FIC_B_. FICI ≤ 0.5 is synergy, 0.5 < FICI ≤1.0 is additive, 1.0 < FICI ≤ 4.0 is indifferent.

### 3.14 Inhibition of inflammatory cytokine production in LPS-stimulated macrophages

**Figure 6:**
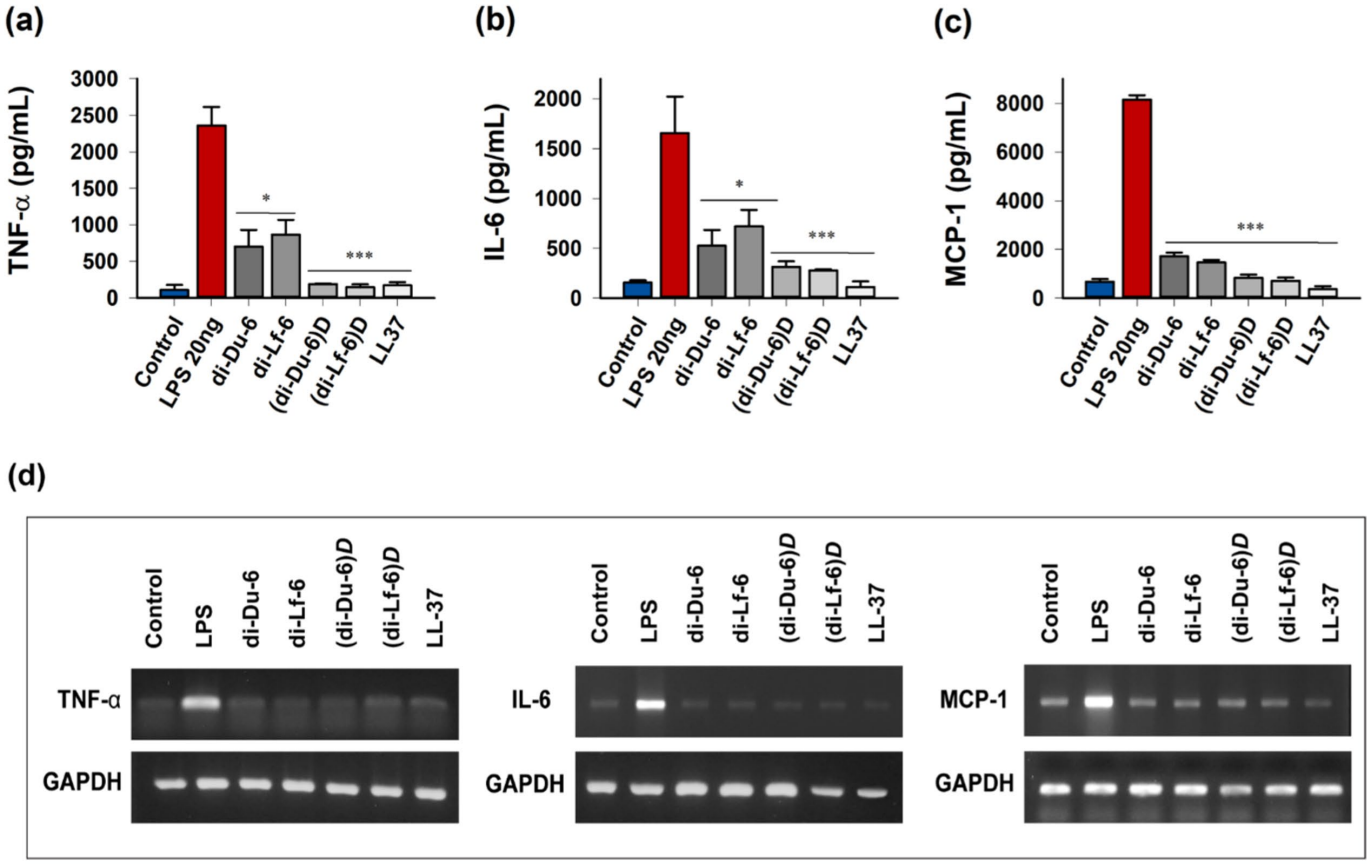
Anti-inflammatory activities of di-Du-6, di-Lf-6, (di-Du-6)*_D,_* and (di-Lf-6)*_D_* in RAW 264.7 murine macrophages. Inhibition of LPS-stimulated production of the inflammatory cytokine **(a)** TNF*-α*, **(b)** IL-6, and **(c)** MCP-1 in RAW 264.7 cells stimulated by LPS. Peptides were administered at a concentration of 8 μg/mL. Data represent the mean ± standard error of the mean (SEM) from at least three independent experiments. Significance is indicated as * for *p* < 0.05, ** for *p* < 0.01, *** for *p* < 0.001. **(d)** Effects of the peptides on the mRNA expression levels of TNF-α, IL-6, and MCP-1 in LPS-stimulated RAW264.7 cells. RAW264.7 cells (5 × 10^5^ cells/well) were incubated with the peptides (8 μg/mL) in the presence of LPS (20 ng/mL) for 3 h. Total RNA was isolated and analyzed to determine the mRNA levels of TNF-α, IL-6, and MCP-1 using reverse transcription-polymerase chain reaction (RT-PCR).

The anti-inflammatory potential of dimeric peptides was assessed by examining their effects on pro-inflammatory cytokine production in lipopolysaccharide (LPS)-stimulated RAW 264.7 murine macrophages. Following LPS stimulation, cells were treated with peptides at sub-cytotoxic concentrations, and cytokine levels were quantified using enzyme-linked immunosorbent assay (ELISA). All dimeric peptides exhibited strong inhibitory effects on LPS-induced cytokine production and expression at 8 μg/mL (Figure 6). TNF-α secretion was significantly reduced, with the D-enantiomeric peptides [(di-Du-6)*_D_* and (di-Lf-6)*_D_*] achieving approximately 80% inhibition. In comparison, the L-form peptides (di-Du-6 and di-Lf-6) showed a 70–75% reduction at the same concentration (Figure 6a). IL-6 production was similarly suppressed, with all peptides reducing IL-6 levels by 60–80% (Figure 6b). MCP-1, a key chemokine involved in the recruitment of inflammatory cells, was effectively inhibited by all dimeric constructs. The D-form peptides demonstrated slightly greater efficacy, with (di-Du-6)*_D_* and (di-Lf-6)*_D_* achieving 85 and 80% inhibition of MCP-1, respectively (Figure 6c). In line with the suppression of pro-inflammatory cytokine production, dimeric peptides treatment also inhibited the expression of TNF-*α*, IL-6, and MCP-1 mRNA in LPS-induced RAW264.7 cells (Figure 6d). Notably, these anti-inflammatory effects were observed at concentrations well below the cytotoxic threshold, confirming that cytokine suppression was due to specific immunomodulatory activity rather than nonspecific cytotoxicity.

### 3.15 Mechanism of anti-inflammatory activity

#### Direct binding and neutralization of LPS

**Figure 7:**
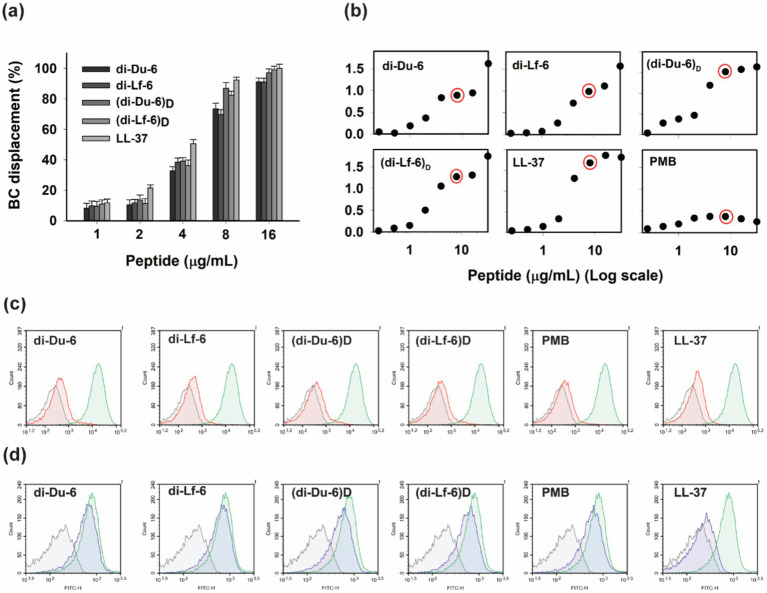
**(a)** Dose-dependent displacement of BODIPY-TR-cadaverine from *E. coli* O111:B4 LPS by peptides and LL-37. **(b)** Dissociation of *E. coli* O111:B4 FITC-LPS aggregates (1 μg/mL), peptides, LL-37, and PMB. Fluorescence intensity was plotted against peptide concentration, with increased emission indicating reduced self-quenching due to disaggregation. Red circle marks peptide concentration of 8 μg/mL. **(c)** Effect of AMPs on the binding of LPS to RAW264.7 macrophage cells. RAW264.7 cells (5 × 10^5^ cells/mL) were incubated with FITC-LPS (1 μg/mL) in the absence (green line) or presence (red line) of peptides (4 μM) for 1 h. The cells were washed, and the binding of FITC-LPS to the macrophages was analyzed using a FACS Calibur flow cytometer. **(d)** Effect of peptides on the binding of LPS to RAW264.7 macrophage cells that were pre-incubated with FITC-LPS. RAW264.7 (5 × 10^5^ cells/mL) cells were pre-incubated with FITC-LPS (1 μg/mL) for 1 h in the absence of peptides (green line). The cells were washed and treated with peptides for an additional 1 h. The FITC-LPS that remained bound to the cells was analyzed by flow cytometry (blue line). The background was determined by recording the fluorescence of the untreated macrophages (black line).

To determine whether the observed anti-inflammatory effects were attributable to direct neutralization of LPS, a BODIPY-TR-cadaverine (BC) displacement assay was conducted. BC selectively binds to the lipid A component of LPS, resulting in fluorescence quenching. When an anti-inflammatory peptide displaces BC, fluorescence is restored, thereby serving as a proxy for LPS-binding affinity (Morrison and Jacobs, 1976). All dimeric peptides demonstrated dose-dependent displacement of BC from *E. coli* LPS, indicating direct binding interactions (Figure 7a). Notably, the D-enantiomeric forms exhibited enhanced LPS-binding capacity: (di-Du-6)*_D_* and (di-Lf-6)*_D_* achieved 90 and 85% BC displacement, respectively, at a concentration of 8 μg/mL. These binding affinities were comparable to those of LL-37, a well-characterized AMP known for its LPS-neutralizing properties. Polymyxin B (PMB) was used as a positive control to quantify the percentage of BC displacement.

#### Dissociation of FITC-LPS aggregates

The capacity of peptides to disaggregate LPS complexes was assessed using fluorescein isothiocyanate (FITC)-conjugated LPS, which exhibits self-quenched fluorescence when aggregated. Peptide-mediated disaggregation leads to fluorescence dequenching, serving as an indicator of LPS-disrupting activity. All dimeric peptides elicited significant, dose-dependent increases in FITC-LPS fluorescence, with detectable activity emerging above 4 μg/mL and maximal disaggregation observed at 16 μg/mL (Figure 7b). LL-37 exhibited comparable disaggregation efficacy. In contrast, PMB showed limited LPS-disrupting activity, inducing only a modest fluorescence increase at 2 μg/mL, which declined at higher concentrations, suggesting minimal disruption of LPS aggregates. These results indicate that the dimeric peptides effectively monomerize LPS aggregates, a mechanism that likely contributes to their LPS-neutralizing properties.

#### Inhibition of LPS-macrophage interactions

To evaluate the impact of peptides on LPS–macrophage binding, flow cytometric analysis was performed. Pre-incubation of FITC-labeled LPS (1 μg/mL) with dimeric peptides (8 μg/mL) significantly reduced cell-associated fluorescence compared to untreated controls, as evidenced by histogram shifts indicating diminished receptor binding (Figure 7c). This inhibitory effect was comparable to that observed with PMB, suggesting that the peptides effectively sequester LPS and prevent its interaction with macrophage receptors. The ability of peptides to inhibit FITC-LPS binding to RAW264.7 cells highlights their potential to block a key inflammatory trigger.

However, when macrophages were pre-treated with FITC-LPS to allow receptor engagement, subsequent peptide administration failed to displace receptor-bound LPS. Under identical conditions, LL-37 removed approximately 50% of receptor-associated LPS, whereas PMB and the dimeric peptides removed less than 10% (Figure 7d). These findings suggest that although dimeric peptides effectively neutralize free LPS via direct binding, they are unable to dislodge LPS already bound to cell surface receptors such as CD14 or TLR4 (Figure 7d) (Jantaruk et al., 2012; Domingues et al., 2012).

## 4 Discussion

The escalating global crisis of antimicrobial resistance demands innovative therapeutic strategies that not only address the limitations of conventional antibiotics but also maintain safety profiles suitable for clinical use (Naghavi et al., 2024). AMPs represent a promising alternative due to their broad-spectrum activity and distinctive mechanisms of action, such as membrane disruption, which help mitigate the emergence of resistance. However, the clinical application of natural AMPs is frequently challenged by their vulnerability to proteolytic degradation and potential cytotoxic effects on mammalian cells (Zasloff, 2002). This study presents a systematic approach to AMP design using lysine-based dendritic architectures, uncovering key structure–activity relationships that enhance therapeutic efficacy while minimizing cytotoxicity.

The branched peptides developed in this study were inspired by specific natural hexapeptide motifs to enhance both antimicrobial potency and therapeutic potential. The Du-6 motif (RKWKRW) originates from the C-terminal residues of dCATH12-5, a duck cathelicidin AMP analog previously identified for its membrane-disruptive antimicrobial activity and endotoxin-neutralizing capabilities (Kumar and Shin, 2020). Likewise, the Lf-6 motif (RRWQWR) is derived from the core helical region of bovine lactoferricin B (LfcinB4-9), recognized for its amphipathic structure and bactericidal efficacy in micellar environments (Schibli et al., 1999). These motifs were strategically chosen for their high content of arginine (Arg) and tryptophan (Trp), which promote electrostatic interactions with anionic bacterial membranes and facilitate hydrophobic insertion, respectively (Chan et al., 2006).

Lysine-based branching was employed to harness multivalency, thereby increasing local charge density and membrane affinity while simultaneously enhancing proteolytic resistance through steric hindrance, an approach supported by previous dendritic peptide designs (Paul et al., 2024; Sheard et al., 2021). In all constructs, β-alanine was added at the C-terminus to enhance flexibility, and amidation was performed to neutralize the terminal carboxyl group. This design strategy enabled precise modulation of net positive charge while preserving the essential pharmacophoric features required for antimicrobial activity (Liu et al., 2010; Klajnert et al., 2006), consistent with established strategies to reduce peptide aggregation and enhance target selectivity (Han et al., 2021). This design framework facilitated a systematic evolution from inactive monomers to optimally active dimers, and ultimately to highly potent yet cytotoxic tetramers, offering valuable insights into structure–activity relationships.

Monomeric peptides (Du-6 and Lf-6) exhibited negligible antimicrobial activity despite containing key cationic and hydrophobic residues. This observation aligns with previous findings that short peptides often require elevated local concentrations or multivalent architectures to reach membrane-disruptive thresholds (Dartois, 2005). In contrast, multimerization markedly improved antimicrobial efficacy, with dimeric and tetrameric constructs achieving geometric mean (GM) MICs of 11–18 μg/mL against both Gram-positive and Gram-negative bacteria, including ESKAPE pathogens (Figure 1). This enhancement is attributed to increased net charge (+8 to +20) and hydrophobicity, which promote stronger membrane interactions, as shown by blue shifts in tryptophan fluorescence within bacterial-mimicking PE/PG vesicles (Figure 2) (Huan et al., 2020). Dimeric peptides demonstrated optimal therapeutic performance, with therapeutic indices (TI) ranging from 30 to 40 substantially surpassing those of natural AMPs such as LL-37 (TI ≈ 10–15) and melittin (TI < 1) (Zasloff, 2002). Although tetrameric peptides displayed superior antimicrobial potency, their therapeutic indices were significantly reduced due to elevated cytotoxicity. This decline is primarily linked to increased hydrophobicity and higher tryptophan content; each tetramer contains eight Trp residues compared to four in dimers, resulting in greater hydrophobic character, as reflected by prolonged HPLC retention times. Prior studies have shown that excessive hydrophobicity in AMPs leads to non-selective membrane disruption and diminished specificity (Chang et al., 2017; Wieprecht, 1997). Our fluorescence analyses offer mechanistic insights into this phenomenon. The pronounced blue shift in *λ*_max_ of tryptophan fluorescence and reduced acrylamide quenching observed for tetramers in PC/Chol SUVs (Figure 2) suggest deeper insertion and stronger interaction with zwitterionic, eukaryotic-like membranes. This enhanced membrane perturbation correlates with increased hemolytic activity and cytotoxicity, thereby lowering the therapeutic index (Laverty and Gilmore, 2014). In contrast, dimers exhibited a more modest blue shift and effective acrylamide quenching, indicating superficial membrane interaction and contributing to their reduced toxicity and improved selectivity. These findings are consistent with previous reports that Trp-rich peptides often display heightened cytotoxicity due to excessive hydrophobicity and indiscriminate disruption of mammalian membranes (Yang et al., 2025; Yin et al., 2012; Paul et al., 2024).

Given their optimal balance between antimicrobial efficacy and low cytotoxicity, the dimeric peptides di-Du-6 and di-Lf-6 were selected as lead candidates for further optimization. This selection provided the rationale for synthesizing D-enantiomeric dimers, designed to overcome the proteolytic instability of L-form peptides while retaining their high therapeutic potential. The resulting D-amino acid-substituted peptides, (di-Du-6)*_D_* and (di-Lf-6)*_D_*, exhibited complete resistance to tryptic degradation (Figure 3) and demonstrated exceptional stability under physiological salt conditions and in human serum (Table 2). This enhanced stability is attributed to the mirrored chirality of D-amino acids, which makes them unrecognizable to proteases that specifically target naturally occurring L-amino acids. As a result, their biological half-life is significantly extended (Kondejewski et al., 1999; Wade et al., 1990).

Importantly, this improvement in stability did not compromise their potent antimicrobial activity. The preservation of key physicochemical properties, such as charge and amphipathicity, ensures that the stereochemistry of the amino acids does not substantially affect their membrane-disrupting mechanism of action (Javadpour et al., 2009; Luo and Song, 2021). Mechanistic assays confirmed that membrane permeabilization is the primary mode of action. The dimers induced rapid membrane depolarization (diSC_3_-5 release), disrupted the outer membrane (NPN uptake), and caused significant loss of membrane integrity (PI influx >75%), closely resembling the activity of melittin (Figure 4). Notably, the D-enantiomers exhibited enhanced permeabilization, likely due to their increased stability. These observations are consistent with previous studies (Luo and Song, 2021), which indicate that cationic AMPs eliminate bacteria by compromising membrane integrity, although their selectivity for microbial versus host membranes may vary.

Beyond their direct antimicrobial activity, dimeric peptides exhibited strong antibiofilm effects against MDRPA, a clinically significant biofilm-forming pathogen (Flemming et al., 2016; Rossi et al., 2021; Malhotra et al., 2019). Both L- and D-form dimers effectively inhibited biofilm formation and disrupted established biofilms at concentrations only 1–2 times higher than the planktonic MICs, with D-form analogs exhibiting greater potency (Figure 5). This strong antibiofilm activity addresses a critical clinical challenge, as biofilms can enhance antibiotic resistance by up to 1,000-fold and play a major role in the persistence of chronic infections (De La Fuente-Núñez et al., 2012). Furthermore, synergistic interactions with conventional antibiotics, as indicated by FICIs ≤ 0.5 for chloramphenicol and ciprofloxacin, highlight the potential of combination therapies to lower effective doses and reduce the emergence of resistance (Sharma et al., 2019). In addition to their potent antimicrobial properties, the dimeric peptides exhibited marked anti-inflammatory activity in LPS-stimulated macrophages (Figure 6). LPS induces a strong inflammatory response by binding to Toll-like receptor 4 (TLR4) on immune cells, thereby triggering downstream signaling cascades and promoting the release of pro-inflammatory cytokines (Akira and Takeda, 2004). ELISA and RT-PCR assays revealed that both L- and D-form dimers significantly suppressed the production and expression of key pro-inflammatory cytokines, including TNF-α, IL-6, and MCP-1 at a relatively low concentration (8 μg/mL) (Figure 6). Given the central role of these cytokines in initiating and amplifying septic inflammation, their inhibition suggests that the peptides may not only combat pathogens directly but also modulate host inflammatory responses that contribute to disease severity (Rosenfeld and Shai, 2006).

Mechanistic assays revealed that the immunomodulatory activity of the dimeric peptides originates from their strong ability to bind and neutralize LPS, a major endotoxin produced by Gram-negative bacteria. The BODIPY-TR-cadaverine displacement assay confirmed that the dimers directly interact with the negatively charged lipid A moiety of LPS, displacing fluorescent probes in a concentration-dependent manner. Direct binding to LPS constitutes a well-established mechanism through which cationic AMPs, such as LL-37 and PMB, exert their anti-inflammatory and endotoxin-neutralizing activities (Rosenfeld and Shai, 2006; Scott et al., 2000). Notably, our D-form dimers achieved displacement efficiencies comparable to LL-37, underscoring their potential clinical relevance.

The LPS engages macrophage surface receptors via LPS-binding protein (LBP), a serum component that facilitates this interaction (Scott et al., 2000). By sequestering LPS, the peptides effectively prevent its interaction with host immune receptors, thereby blocking the initiation of the inflammatory cascade and the subsequent release of cytokines such as TNF-α, IL-6, and MCP-1 (Cross and Opal, 1995). In addition, the dimers disrupted LPS aggregates, converting them into monomeric forms with reduced pro-inflammatory potential (Figure 7b). This activity is particularly important, as aggregated LPS strongly activates Toll-like receptor 4 (TLR4) signaling in macrophages, leading to excessive cytokine production (Erridge et al., 2002). By monomerizing these aggregates, the dimers attenuate receptor activation and downstream inflammatory responses.

Furthermore, flow cytometry analysis demonstrated that both L- and D-form dimers inhibited the binding of FITC-labeled LPS to macrophage surfaces, likely by competitively binding to LPS and preventing its interaction with LPS-binding protein (LBP) and CD14 co-receptors (Scott et al., 2000; Nagaoka, 2002). This mechanism mirrors that of PMB, which neutralizes LPS by binding to its lipid A moiety (Morrison and Jacobs, 1976), and is consistent with peptides interfering with LPS–LBP interactions, thereby preventing macrophage activation and subsequent cytokine release (Scott et al., 2000). However, once LPS had already bound to macrophage receptors, the peptides were unable to displace it. This limitation was also observed with PMB (Domingues et al., 2012). These findings suggest that the peptides are most effective in preventing the initiation of LPS-driven inflammation, rather than reversing established endotoxin-receptor interactions.

Collectively, these mechanistic insights demonstrate that the anti-inflammatory activity of dimeric peptides is mediated through direct LPS neutralization, including high-affinity binding to lipid A, disruption of LPS aggregates, and inhibition of interactions between LPS and cell surface receptors, thereby preventing activation of the inflammatory cascade (Rosenfeld et al., 2006; Nagaoka, 2002). These findings establish the peptides as dual-function agents that exert bactericidal effects through membrane disruption while simultaneously attenuating septic inflammation by binding, monomerizing, and sequestering LPS. The ability of D-form dimers to preserve and even enhance these functions is particularly encouraging, given that proteolytic stability is essential for systemic applications in treating infections and sepsis. This aligns with the growing consensus that successful antimicrobial therapeutics must target the host-pathogen interface, not merely the pathogen, to reduce microbial burden and mitigate host-driven inflammatory damage (Hancock and Sahl, 2006; Mookherjee and Hancock, 2007).

The dimeric peptides exhibit a unified mechanistic framework, driven by lysine-branching-induced multivalency, that orchestrates extracellular membrane disruption and downstream immunomodulation across bactericidal, antibiofilm, LPS-neutralizing, and anti-inflammatory activities. Core to this mechanism is a two-step process: rapid electrostatic recruitment to anionic microbial surfaces, followed by tryptophan-mediated hydrophobic insertion and permeabilization (Chan et al., 2006; Zhang et al., 2021). Spatiotemporal nuances differentiate targets: Planktonic killing involves swift outer/inner membrane breach (Figure 4), while biofilms demand sustained penetration of extracellular polymeric substances over hours (Figure 5) (Flemming et al., 2016). The high cationic charge allows for immediate extracellular sequestration of the LPS through direct binding to Lipid A (Figure 7a) and dissociation of inflammatory LPS aggregates (Figure 7b). This upstream blockade prevents the LPS from engaging macrophage receptors, thereby suppressing the production of cytokines (Figure 6). The D-enantiomeric dimers retain all efficacy while gaining complete proteolytic stability, significantly extending their effective spatiotemporal window. Compared to LL-37, our branched design confers superior selectivity by confining action to extracellular interfaces (modest PC/Chol blue shift; Figure 2a), minimizing the cytotoxicity associated with deeper eukaryotic membrane penetration. This integrated framework validates our *de novo* design, positioning these multifunctional peptides as promising candidates for treating MDR infections and sepsis.

In conclusion, this study presents a rational design strategy for developing highly potent and selective branched peptides. The dimeric constructs, especially the D-enantiomeric analogs, represent a significant advancement in overcoming the conventional limitations of AMPs, offering a compelling combination of broad-spectrum antimicrobial activity, minimal host cell toxicity, exceptional proteolytic stability, and pronounced anti-inflammatory effects. These multifunctional peptides hold strong potential as next-generation anti-infective therapeutics, particularly in the context of rising antimicrobial resistance.

## 5 Conclusion

This study highlights the successful design and evaluation of lysine-branched dendritic AMPs derived from Du-6 and Lf-6 motifs. The dimeric constructs demonstrated enhanced broad-spectrum activity against ESKAPE pathogens, along with high therapeutic indices and multifunctional properties. Incorporation of D-enantiomers provided complete proteolytic stability and resilience under physiological conditions. Mechanistic investigations confirmed membrane disruption as the primary mode of antibacterial action. Additionally, these peptides exhibited strong antibiofilm activity, synergistic effects with conventional antibiotics, and anti-inflammatory capabilities through direct neutralization of LPS, effectively addressing major challenges in antimicrobial resistance and inflammation. Collectively, these findings position the branched dimers as promising candidates for next-generation anti-infective therapies, meriting further preclinical and *in vivo* validation.

## Data Availability

The original contributions presented in the study are included in the article/Supplementary material, further inquiries can be directed to the corresponding authors.
