# Supplementary material for: Development of lysine-branched dendrimeric antimicrobial peptides targeting ESKAPE pathogens: broad-spectrum activity, biofilm eradication, and endotoxin neutralization
Source: Front Microbiol. 2026 Feb 23;16:1702629. doi: 10.3389/fmicb.2025.1702629 (PMC12967992; doi:10.3389/fmicb.2025.1702629)

Development of Lysine-Branched Dendrimeric Antimicrobial Peptides Targeting ESKAPE Pathogens: Broad-Spectrum Activity, Biofilm Eradication, and Endotoxin Neutralization

S. Dinesh Kumar^1,†^, Eun Young Kim^1,†^, Naveen Kumar Radhakrishnan^2^, Byambasuren Ganbaatar^3^, Chul Won Lee^3^, Sungtae Yang^4,*^ and Song Yub Shin^1,*^

*^1^Department of Cellular & Molecular Medicine, School of Medicine, Chosun University, Gwangju, 61452, Republic of Korea.*

*^2^Department of Biomedical Sciences, School of Medicine, Chosun University, Gwangju 61452, Republic of Korea.*

*^3^Department of Chemistry, Chonnam National University, Gwangju 61186, Republic of Korea*

*^4^Department of Microbiology, School of Medicine and Institute of Well-Aging Medicare & CSU GLAMP Project Group, Chosun University, Gwangju 61452, Republic of Korea*

^†^ These authors contributed equally to this work.

***Correspondences:**

Sungtae Yang, E-mail: [styang@chosun.ac.kr](mailto:styang@chosun.ac.kr)

Song Yub Shin, E-mail: [syshin@chosun.ac.kr](mailto:syshin@chosun.ac.kr)

**Contents:**

1. Materials and methods ----------------------------------------------------------------------------- S3
2. Table S1. Antimicrobial activities of D-form peptides ---------------------------------------- S12
3. Figure S1. RP-HPLC profiles of the synthetic peptides --------------------------------------- S13
4. Figure S2. ESI-MS ionization spectra of the synthetic peptides ----------------------------- S14

**1. MATERIALS AND METHODS**

**1.1. Materials**

Rink amide-methylbenzhydrylamine (MBHA) resin, 9- fluorenylmethoxycarbonyl (Fmoc) protected amino acids, and other chemicals and solvents used for peptide synthesis were bought from Novabiochem (La Jolla, CA, USA). LPS purified from *Escherichia coli* O111:B4, 2,2,2-trifluoroethanol (TFE), 3-(4,5-dimethylthiazol-2-yl)-2,5-diphenyl-2H-tetrazolium bromide (MTT), N-phenyl-1-naphthylamine (NPN), 3,3’-dipropylthiadicarbocyanine (diSC_3_-5), propidium iodide, proteolytic enzyme (trypsin) and all antibiotics, were supplied from Sigma-Aldrich (St. Louis, MO, USA). HyClone Dulbecco’s modified Eagle’s medium (DMEM) and fetal bovine serum (FBS) were obtained from SeouLin Bioscience (Seoul, Korea). Buffers were prepared using Milli-Q ultrapure water (Merck Millipore, Billerica, MA, USA). All reagents were of analytical grade. The TNF-*α* ELISA kit was procured from R&D Systems (Minneapolis, MN, USA). All buffers were prepared using Milli-Q ultrapure water (Merck Millipore, USA). RAW264.7 (mouse macrophage) cells were purchased from the American Type Culture Collection (Manassas, VA).

**1.2. Bacterial strains**

A total of 12 microbial strains were used in this study. Three Gram-positive strains (*Staphylococcus epidermidis* (KCTC 1917), *Staphylococcus aureus* (KCTC 1621), and *Bacillus subtilis* (KCTC 3068)) and three Gram-negative strains (*Escherichia coli* (KCTC 1682), *Pseudomonas aeruginosa* (KCTC 1637), and *Salmonella typhimurium* (KCTC 1926)) were obtained from the Korean Collection for Type Cultures (KCTC) at the Korea Research Institute of Bioscience and Biotechnology (KRIBB). Multidrug-resistant strains, including *Enterococcus faecium* (MDREC 329-57), *Klebsiella pneumoniae* (MDRKP 328-89), *Acinetobacter baumannii* (MDRAB 329-53), and *Pseudomonas aeruginosa* (MDRPA 321-16), were provided by Chosun University Hospital. Methicillin-resistant *Staphylococcus aureus* (MRSA CCARM 3090) was obtained from the Culture Collection of Antibiotic-Resistant Microbes (CCARM) at Seoul Women’s University. Vancomycin-resistant *Enterococcus faecalis* (VREF) (ATCC 51559) was purchased from the American Type Culture Collection (ATCC).

**1.3. Antimicrobial activity**

Antimicrobial susceptibility of the designed peptides against standard bacteria, drug-resistant bacteria and yeast was studied according to guidelines of clinical and laboratory standards institute (CLSI) (CLSI, 2012 Edition). Prior to assays, all strains were cultured overnight to stationary phase at 37 °C in LB media. The overnight cultures were 10-fold diluted in fresh Muller-Hinton (MH) broth (Difco, USA) and grown for additional few hours at 37 °C and 28 °C to achieve mid-log phase growth. This mid-log phase cultures were diluted with MHB and added to sterile 96-well plates containing two-fold serially diluted peptides (from 2 to 512 *µ*M) in 1:1 ratio to give final cell concentration of 2 ×10^6^ CFU/wells. Melittin treated cells were used as positive control for both bacteria and yeast while untreated cells were used as a negative control. The minimal inhibitory concentration (MIC) was defined as the lowest peptide concentration that causes 100% inhibition of microbial growth after incubation at 37 °C for 24 h. In addition, the MICs of the peptides were also determined in the presence of different salts and human serum. 2 × 10^6^ CFU/mL of *E. coli* (KCTC 1682), and *S. aureus* (KCTC 1621) were treated with peptides in MHB supplemented with different salts at their physiological concentrations (150 mM NaCl or 4.5 mM KCl or 6 *µ*M NH_4_Cl or 1 mM MgCl_2_ or 2.5 mM CaCl_2_) or 20% human serum. Each test was reproduced at least three times using six replicates.

**1.4. Hemolytic activity assay**

The hemolytic activity of peptides was evaluated by measuring the amount of free hemoglobin by the lysis of erythrocytes using sheep red blood cells sRBCs. Fresh sRBCs were washed thrice and resuspended with 1×PBS (pH 7.2), followed by centrifugation for 5 min at 116×g. 4% (w/v) sRBC suspension was incubated with peptide-containing PBS (2-512 *µ*M). After incubation for 1 h at 37 °C, the samples were centrifuged at 1000× g for 5 min. Absorbance of supernatant (hemoglobin) was recorded using a microplate ELISA reader (Bio-Tek Instruments EL800, USA) at 540 nm. As a positive control, 100% hemolysis was induced by treating sRBCs with 0.1% Triton X-100. Melittin was used as a reference peptide. The value for “zero hemolysis” was determined using PBS (Kumar and Shin, 2020).

**1.5. Cytotoxicity assay**

To determine the cytotoxicity of the peptides, we used the MTT dye reduction assay against RAW 264.7 cells as previously described (Kumar and Shin, 2020). Briefly, the cells (2 × 10^4^ cells/well in DMEM supplemented with 10% FBS) were placed into 96-well plates and incubated for 18-24 h at 37 °C in 5% CO_2_. Peptides were then added to the cells at final concentrations of 1.25-80 *µ*M. After incubation for 24 h, 20 µL MTT (5 mg/mL) reagent was added to each well and incubated for an additional 4 h. The formazan crystals produced were dissolved in dimethyl sulfoxide (DMSO), and the absorbance at 570 nm was measured using a microplate ELISA reader.

**1.6. Preparation of small unilamellar vesicles (SUVs)**

Lipids, including Phosphatidylglycerol (PG), Phosphatidylcholine (PC), Phosphatidylethanolamine (PE), and cholesterol, were obtained from Sigma-Aldrich (St. Louis, MO, USA). Two types of liposomes with different lipid ratios were prepared as follows: PC/cholesterol (10:1, w/w) to mimic the human erythrocyte cell membrane; PE/PG (7:3, w/w) to mimic the bacterial membrane. For small unilamellar vesicles (SUVs) preparations, lipids were dissolved in chloroform, the organic solvent was dried by rotary evaporation to form a thin film on the sides of the round-bottomed flask, and then lyophilized overnight. Dried thin films were resuspended in 10 mM Tris-HCl buffer (10mM Tris 150mM NaCl, 0.1mM EDTA, pH 7.4) by vortexing. The lipid dispersions were sonicated in ice water for 20 min using an ultrasonic cleaner until the solutions clarified (De Kroon, et al., 1990).

**1.7. Intrinsic Tryptophan Acrylamide Quenching Assay**

For the fluorescence quenching experiment, acrylamide was used as the quencher, and the fluorescence measurements were recorded using the RF-5301 PC fluorescence spectrophotometer (Shimadzu Scientific Instruments, Kyoto, Japan). To reduce the absorbance of acrylamide, Trp was excited at 295 nm and emission at 340 nm (slit width 5 nm). Trp fluorescence was quenched by the titration of acrylamide from a 4 M stock solution to the final concentration of 0.4 M in the presence of PE/PG and PC/cholesterol SUVs at peptide/lipid molar ratio of 1:200. The effect of acrylamide on the fluorescence of each peptide was analyzed by a Stern–Volmer equation: F_0_/F = 1 + K_SV_ [Q], where F_0_ and F are the fluorescence values of the peptide in the absence or the presence of acrylamide, respectively, K_SV_ represents the Stern-Volmer quenching constant, and [Q] represents the concentration of acrylamide (Zhu et al., 2006).

**1.8. Trypsin digestion**

Peptides were incubated with trypsin (2 μg/mL) in 50 mM Tris buffer with 5 mM CaCl_2_ at pH 7.4 and 25 °C. The peptide cleavage after fixed time intervals (0, 30, 60, 120 min and 24 h) was monitored by analytical RP-HPLC using a C_18_ reverse-phase column (4.6 mm × 250 mm; Vydac) with a linear gradient of 0.05% aqueous TFA and 0.05% TFA in acetonitrile. The absorbance was measured at 215 nm. Digestion was determined by quantifying the area under the peak of native peptides at the respective retention times in the HPLC chromatograms (Kim et al., 2023).

**1.9. Bacterial membrane depolarization assay**

The ability of peptides to depolarize the intact *S. aureus* cytoplasmic membrane was determined using the membrane potential-sensitive cationic probe DiSC_3_-5. Briefly, mid-log phase *S. aureus* (KCTC 1621) cells were harvested and washed three times with 5 mM HEPES buffer (pH 7.4, containing 20 mM glucose and 100 mM KCl) and the cells were resuspended to an *A*_600nm_ of 0.05 in same buffer and incubated with 20 nM diSC_3_-5 (Sigma, USA) until a stable reduction of the fluorescence was observed, implying the complete intake of dye into cytoplasm. The peptides (2× MIC) were then added to bacterial suspension, and changes in fluorescence intensity were recorded (excitation λ = 622 nm, emission λ = 670 nm) using a RF-5301 PC spectrofluorophotometer (Shimadzu, Japan). 0.1 % Triton X-100 was added to completely dissipate the membrane potential. When added to the bacteria, diSC_3_-5 is influxed onto the cytoplasmic membrane and its fluorescence is self-quenched under the influence of membrane potential. Upon disruption of the cytoplasmic membrane, diSC_3_-5 is released into the buffer, resulting in an increase in fluorescence (Kim et al., 2023).

**1.10. Flow cytometry analysis**

The bacterial membrane integrity was assessed by flow cytometry as previously described (Kim et al., 2023). Briefly, mid-log phase of *E. coli* (KCTC 1682) were washed thrice and diluted to 1 × 10^5^ CFU/mL in 1× PBS. The peptides (2 × MIC) were added to the bacterial cell suspension at fixed Propidium iodide (PI) concentration of (10 *µ*g/mL) and incubated for 1h at 37 °C with agitation. The unbound dye are washed with PBS and flow cytometry data were recorded using fluorescence-activated cell sorter (FACS Calibur, Beckman Coulter Inc., USA) at an excitation wavelength of 488 nm.

**1.11. Outer membrane permeability assay**

The hydrophobic fluorescent probe NPN (1-N-phenylnaphthylamine) was used to determine outer membrane permeability of gram-negative bacteria. Briefly, mid-log phase of *E. coli* (KCTC 1682) cells were washed thrice in 5 mM HEPES buffer (pH 7.4, containing 20 mM glucose and 5 mM KCN) and diluted to an *A*_600nm_ of 0.05 in same buffer. 1mM stock solution of NPN was prepared by dissolving in acetone. From the stock solution 30*μ*L was added to bacterial suspension to reach final concentration of 10 *μ*M and the background fluorescence was recorded (excitation λ = 350 nm, emission λ = 420 nm) until the stable fluorescence was achieved. Increasing concentrations of peptides was then added and fluorescence was recorded with respect to time until there was no further increase in fluorescence (Kumar and Shin, 2020).

**1.12. Biofilm inhibition assay (MBIC)**

Briefly, 1×10^6^ CFU/200 μl of MDRPA (321-16) in Mueller Hinton broth (MHB) with 0.2% glucose were incubated at 37 °C for 24 h in 96-well microtiter plates, with or without peptides. After the incubation, the planktonic bacteria were removed by washing three times with PBS solution. Afterwards, 99% methanol was added and fixed for 15 min. After aspiration, the plates were allowed to dry. Dried wells were stained with 100 μl of 0.1% crystal violet for 5 min and excess stain was gently rinsed off with tap water. Stain was resolubilized in 95% ethanol and absorbance at 600 nm was measured. Untreated bacteria were used as the control. All biofilm inhibition assays were performed in triplicates. The concentration of the peptide that completely prevented residual CV staining/biomass was the MBIC (Harrison et al., 2010).

**1.13. Biofilm eradication assay (MBEC)**

Briefly, 1 × 10^6^ CFU/mL of MDRPA (321-16) were suspended in 150 μL of appropriate nutrient media (LB media) and placed in 96-well microtiter plates with peg lids (Innovotech, Edmonton, Canada; product code: 19111) to establish biofilms. Plates were sealed with parafilm and incubated at 37 °C for 24 h in a shaking incubator at 110 rpm. Lids of the plates were then removed, rinsed with 0.01 M PBS, and transferred to sterile 96-well plates containing serial dilutions of the compounds: the final volume with the media was 200 μL/well. Plates were then incubated at 37 °C for 24 h in a shaking incubator at 110 rpm. After 24 h of treatment, the peg lid of each plate was removed, rinsed with buffer, and transferred to a recovery plate containing 200 μL of nutrient media. Recovery plates were thereafter sonicated in a water bath for 10–15 min to dislodge biofilms. Peg lids were removed and plates were incubated overnight (for 24 h) at 37 °C in a shaking incubator at 110 rpm to recover viable bacteria, resulting in turbidity. The MBEC values were recorded as the lowest concentration resulting in eradication of the biofilm (i.e., no turbidity after the final incubation period relative to sterility controls). Experiments were performed in triplicates, and the median value of each experiment was presented (Harrison et al., 2010).

**1.14. Confocal laser scanning microscopy (CLSM)**

MDRPA (321-16) (1×10^6^ CFU/mL) was cultured in 24-well plates containing discs placed in MHB-glucose medium, for 24 h to form biofilms. Discs with planktonic cells were washed with 1× PBS thrice and placed in fresh 24-well plates containing the compounds (MBEC_90_), and plates were incubated for 6 h. Discs were removed, washed twice with 1 × PBS, and concomitantly stained with 6.7 μM SYTO 9 and 40 μM PI. After incubation in the dark at 37 °C for 30 min, planar images of biofilm mass in the discs were visualized using CLSM (Zeiss LSM 710 Meta, ZEISS Microscopy, Jena, Germany), and analyzed using ZEN 2009 Light Edition software (ZEISS Microscopy, Jena, Germany) (Kim et al., 2023).

**1.15. Synergy testing by checkerboard assay**

Initially, 2-fold serial dilutions of each antibiotic and each compound were prepared and added in a 1:1 volume ratio to the wells of a 96-well plate. An equal volume of bacterial solution (100 μL) at ~10^6^ CFU/mL was then seeded into each well. The plates were incubated in a shaking incubator at 37 °C and 200 rpm and read after 72 h. Bacterial growth was assessed visually or spectrophotometrically via OD_600_ readings taken by a microplate ELISA reader (Molecular Devices, Sunnyvale, CA, USA). The broth microdilution checkerboard technique was employed for FIC determination, with the FIC index (FICI) defined as the sum of each MIC of compound-antibiotic combination divided by each individual antimicrobial agent and according to the following equation: FICI = [(MIC of antibiotic in combination) / (MIC of antibiotic alone)] + [(MIC of small molecule in combination) / (MIC of small molecule alone)], where FICI ≤ 0.5 is considered as indicative of synergy; 0.5 < FICI ≤ 1.0 is considered as additive; 1.0 < FICI ≤ 4.0 is considered as indifferent; and FICI > 4.0 is considered as indicative of antagonism (Qu et al., 2017).

**1.16. Quantification of inflammatory cytokine production in LPS-stimulated macrophage cells**

Peptide-induced inhibition of pro-inflammatory cytokines production in LPS-stimulated macrophage cells were measured as previously described (Kumar and Shin, 2020). In brief, RAW 264.7 murine macrophage cells (2×10^6^ cell/mL) were plated and adhered to a 96 well plates (100 *µ*L/well) and stimulated with LPS from *E. coli* O111:B4 (20 ng/mL) in the presence or absence of peptides for 24 h. After 24 h incubation, the culture supernatant was collected for enzyme linked immunosorbent assay (ELISA) to detect the level of inflammatory cytokines TNF-*α*, IL-6, and MCP-1. The nitrite level was determined using Griess reagent (1% sulfanilamide, 0.1% naphthylethylenediamine dihydrochloride and 2% phosphoric acid). Release of pro-inflammatory cytokines was detected using DuoSet ELISA mouse TNF-*α* (R&D Systems, Minneapolis, USA) according to the manufacturer’s protocol.

**1.17. Reverse-transcription polymerase chain reaction (RT-PCR)**

RAW264.7 cells were seeded into 6-well plates at 2 × 106 cells/well and stimulated with E. coli O111:B4 LPS (20 ng/mL) in the presence or absence of peptides. After incubation of 3 h total RNA was extracted using TRIzol® reagent (Invitrogen) and RNA concentration quantified using Nanodrop spectrophotometer (BioDrop, UK). cDNA was synthesized from 2 μg of total RNA using Oligo-d(T)15 primers and PrimeScript Reverse Transcriptase kit (Takara, Japan) according to the manufacturer’s protocol. The PCR amplification was carried out for initial denaturation at 94 °C for 5 min, followed by forty cycles of denaturation at 94 °C for 1 min, annealing at 55 °C for 120 s and extension at 72 °C for 1 min, with a final extension at 72 °C for 5min. The PCR products were separated by electrophoresis and visualized under UV illumination.

**1.18. LPS neutralization assay**

The ability of peptides to bind with LPS was determined using a fluorescent probe BODIPY-TR cadaverine (BC) (Sigma, USA) displacement assay as previously described (Wood et al., 2004). Briefly, LPS from *E. coli* 0111:B4 (25 *µ*g/mL) was incubated with BC (2.5 *µ*g/mL) in a quartz cuvette containing 50 mM Tris buffer (pH 7.4). Increasing concentration of peptides was then added over a 100-second time interval, and changes in fluorescence were recorded (excitation λ = 580 nm, emission λ = 620 nm) using an RF-5301 PC Spectrofluorophotometer (Shimadzu, Japan). The values were converted to %∆F (AU) using the following equation:

*% ∆F (A.U.) = [(F_obs_ – F_0_) / (F_100_ – F_0_)] × 100*

where F_obs_ is the observed fluorescence at a given peptide concentration, F_0_ is the initial fluorescence of BC with LPS in the absence of peptides, and F_100_ is the BC fluorescence with LPS cells upon the addition of 10 mg/mL polymyxin B (a prototype LPS binder) which is used as positive control.

**1.19. Dissociation of LPS-FITC aggregates**

The ability of peptides to disaggregate FITC-LPS oligomers was performed as described previously (Rosenfeld et al., 2006; Haas et al., 2000). Briefly, FITC-LPS (1 *µ*g/mL) was added in a quartz cuvette containing 1× PBS, and background fluorescence was measured at emission of 515 nm using 5301 PC Spectrofluorophotometer. The variations in emission of FITC after the addition of different concentrations of peptides were recorded (excitation *λ* = 488 nm, emission *λ* = 512 nm). The emissions of both PBS and peptides alone were taken as a positive control. Dissociation of the aggregates of FITC-LPS results in an increase in the fluorescence of FITC because of dequenching. The changes in emissions were tracked until the system reached equilibrium.

**1.20. Effect of peptides on LPS binding to macrophages**

The ability of peptides to bind with FITC-conjugated LPS (Sigma, USA) was performed as previously described (Kim et al., 2023; Rosenfeld et al., 2006). Briefly, FITC-LPS (1 *µ*g/mL) was incubated with peptides (10 *µ*M final concentration) for 1 h at 4 °C. Thereafter, 5×10^5^ cells/mL of RAW264.7 macrophage cells were treated with FITC-LPS/peptide mixture and incubated at 37 °C for 30 min. The cells were then washed with ice-cold PBS (pH 7.4) extensively to remove the unbound LPS. The binding of FITC-LPS to RAW264.7 cells was analyzed by measuring median fluorescence intensity using flow cytometry (FACS Calibur, Beckman Coulter Inc., USA). A prototype LPS binding inhibitor, PMB (Polymyxin B), and LL-37 were used as positive controls. Background fluorescence was assessed by using RAW 264.7 cells incubated without FITC-LPS or peptides. Data are from one of five separate experiments.

**1.21. Effect of peptides on receptor-bound LPS**

RAW264.7 macrophage cells (5×10^5^ cells/mL) were incubated for 1h with FITC-LPS (1 *µ*g/mL) in the absence of peptides. The pre-incubated cells were washed three times with 1× PBS to remove unbound LPS. The peptides were then added to the cells and incubated for an additional 1h, and then washed again. The binding of FITC-LPS to RAW264.7 cells after treatment of peptides was analyzed by flow cytometry (Kim et al., 2023; Rosenfeld et al., 2006).

**Table S1.** Antimicrobial activities of d-form analogs (di-Du-6)*_D_* and (di-Lf-6)*_D_* against different microorganisms

| Bacterial strains | MIC *^a^* (*µ*g/mL) | |
| --- | --- | --- |
|  | (di-Du-6)*_D_* | (di-Lf-6)*_D_* |
| Gram-negative bacteria |  |  |
| *Escherichia coli* (KCTC 1682) | 8 | 16 |
| *Pseudomonas aeruginosa* (KCTC 1637) | 16 | 16 |
| *Salmonella typhimurium* (KCTC 1926) | 16 | 16 |
| Gram-positive bacteria |  |  |
| *Staphylococcus aureus* (KCTC 1621) | 8 | 8 |
| *Staphylococcus epidermidis* (KCTC 1917) | 8 | 8 |
| *Bacillus subtilis* (KCTC 3068) | 8 | 8 |
| Multidrug-resistant ESKAPE pathogens |  |  |
| MDREC (329-57) | 16 | 32 |
| MRSA (CCARM 3090) | 16 | 32 |
| MDRKP (328-89) | 16 | 16 |
| MDRAB (329-53) | 32 | 32 |
| MDRPA (321-16) | 32 | 16 |
| VREF (ATCC 51559) | 16 | 16 |

**Figure S1. RP-HPLC profiles of the synthetic peptides**


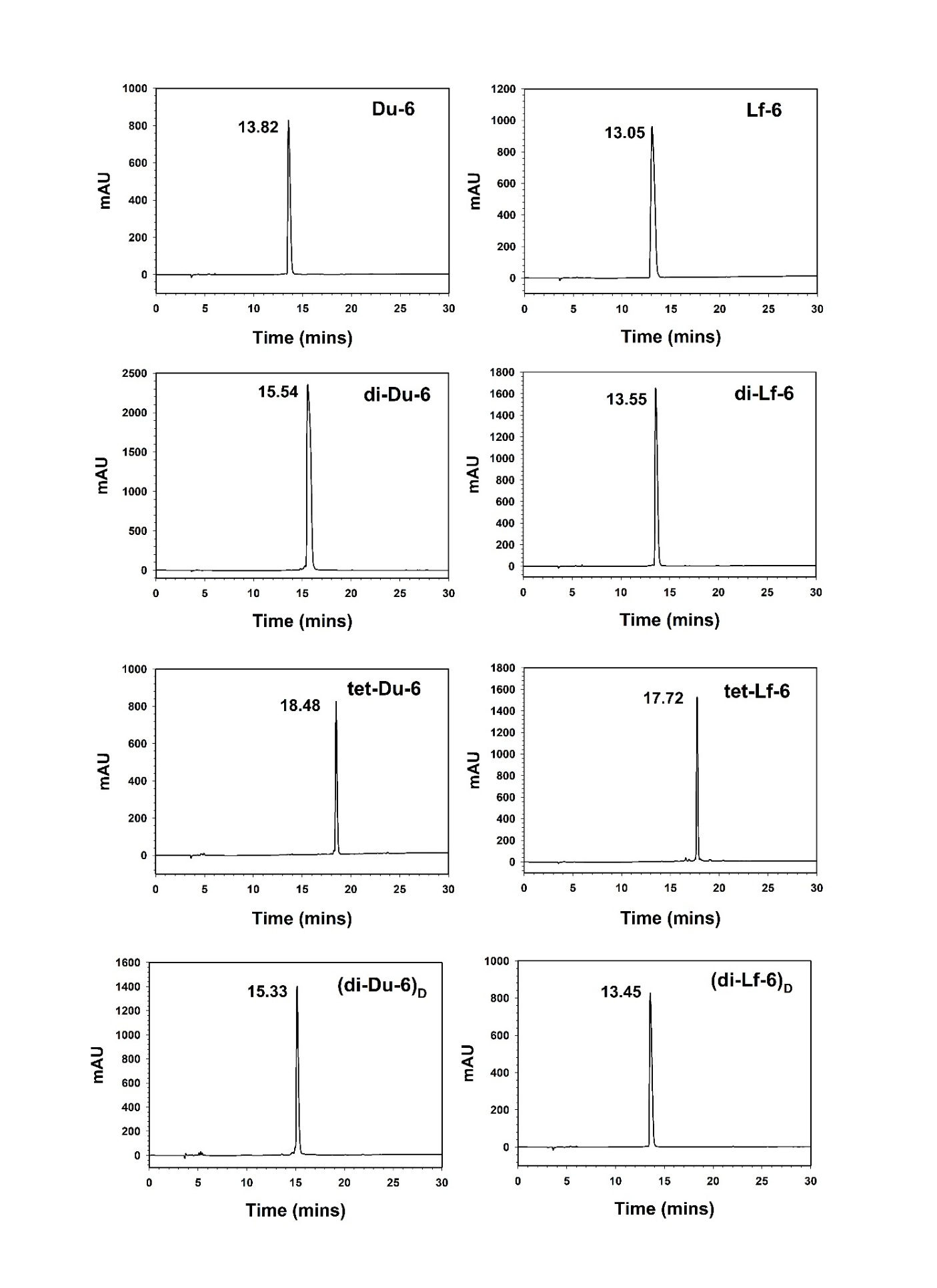


**Figure S2. ESI/MS spectra of the synthetic peptides**


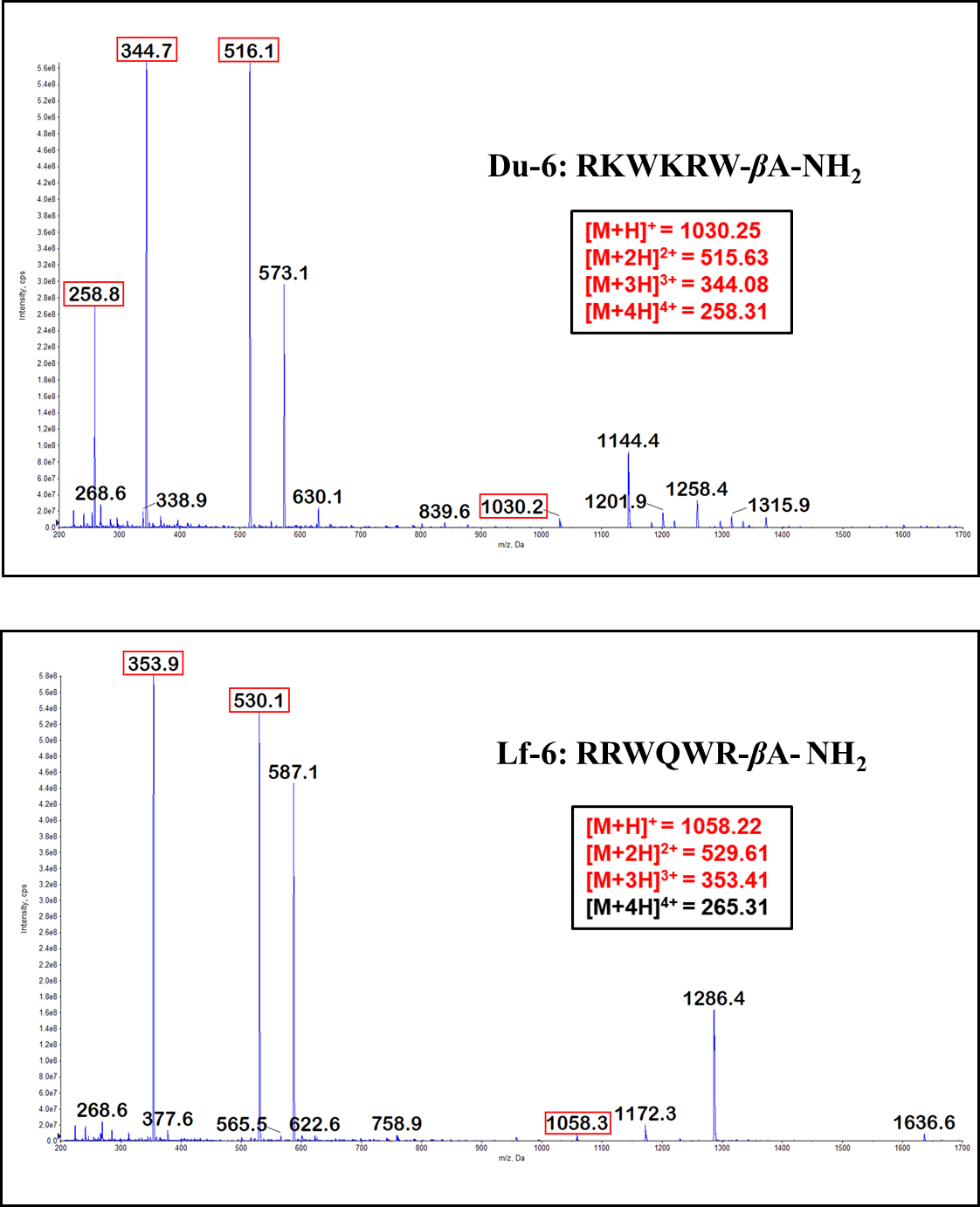


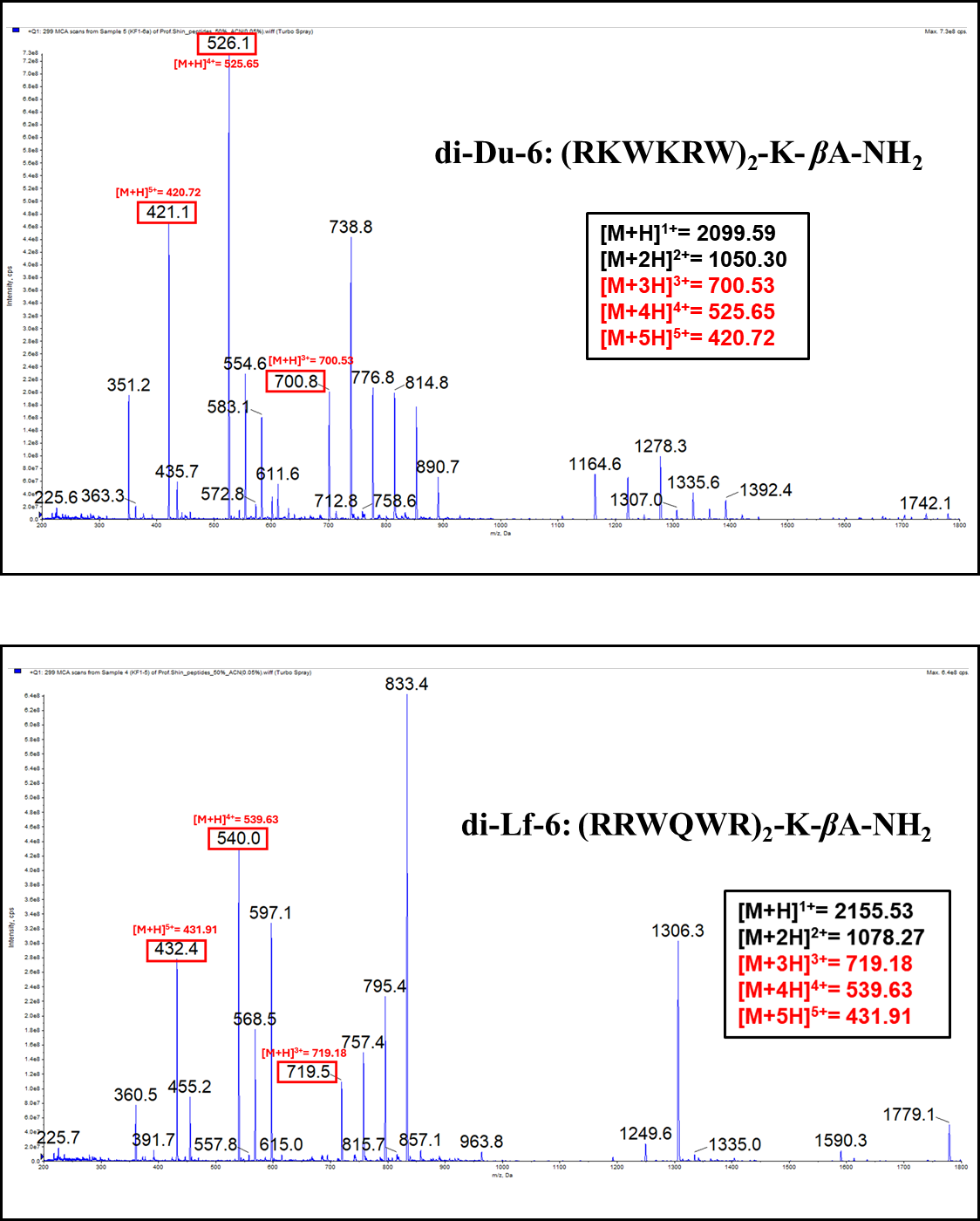


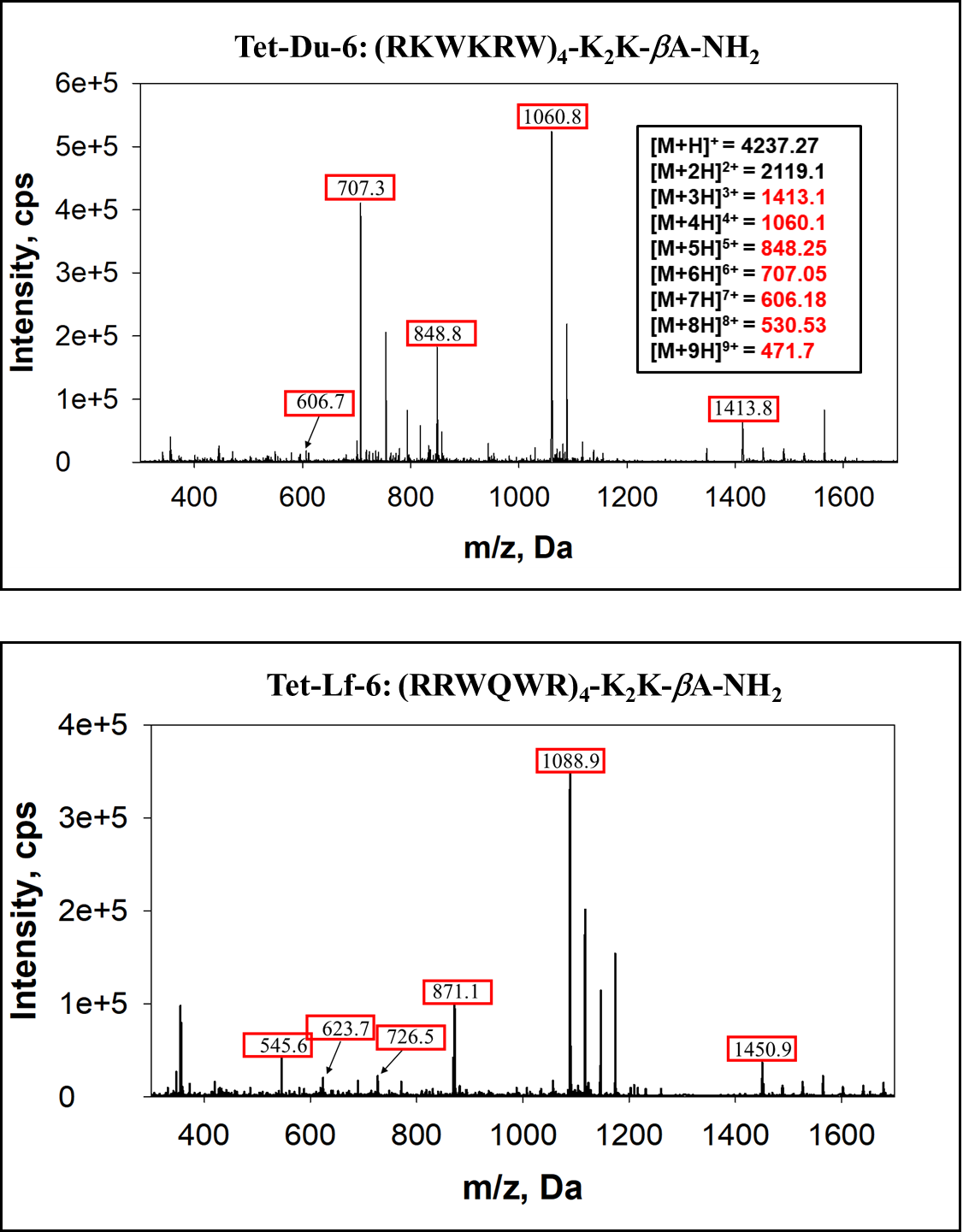

Supplement: Supplementary file 1 [file Data_Sheet_1.docx]
